# Supplementary material for: In vivo assembly enhanced binding effect augments tumor specific ferroptosis therapy
Source: Nat Commun. 2024 Jan 11;15:454. doi: 10.1038/s41467-023-44665-2 (PMC10784468; doi:10.1038/s41467-023-44665-2)
Supplement: Supplementary file 1 — Supplementary information [file 41467_2023_44665_MOESM1_ESM.pdf]

Supplementary Information

In Vivo Assembly Enhanced Binding Effect Augments Tumor Specific Ferroptosis Therapy

Da-Yong Hou,[#,a,b,c] Dong-Bing Cheng,[#,d] Ni-Yuan Zhang,[#,a] Zhi-Jia Wang,[a,b,c] Xing-Jie Hu,[a] Xin Li,[d] Mei-Yu Lv,[b] Xiang-Peng Li,[a,b,c] Ling-Rui Jian,[b,c] Jin-Peng Ma,[b,c] Taolei Sun,[d]* Zeng-Ying Qiao,[a]* Wanhai Xu[b,c]* and Hao Wang[a]*

[a]CAS Key Laboratory for Biomedical Effects of Nanomaterials and Nanosafety, CAS Center for Excellence in Nanoscience, National Center for Nanoscience and Technology (NCNST), Beijing, 100190, China.

[b]NHC and CAMS Key Laboratory of Molecular Probe and Targeted Theranostics, Harbin Medical University, Harbin, 150001, China.

[c]Department of Urology, Harbin Medical University Cancer Hospital, Heilongjiang Key Laboratory of Scientific Research in Urology, Harbin, 150001, China.

[d]School of Chemistry, Chemical Engineering & Life Science, Hubei Key Laboratory of Nanomedicine for Neurodegenerative Diseases, Wuhan University of Technology, No. 122 Luoshi Road, Wuhan, 430070, PR China.

[#]These authors contributed equally: Da-Yong Hou, Dong-Bing Cheng and Ni-Yuan Zhang.

***Corresponding e-mail address**: suntl@whut.edu.cn, qiaozy@nanoctr.cn, xuwanhai@hrbmu.edu.cn and wanghao@nanoctr.cn

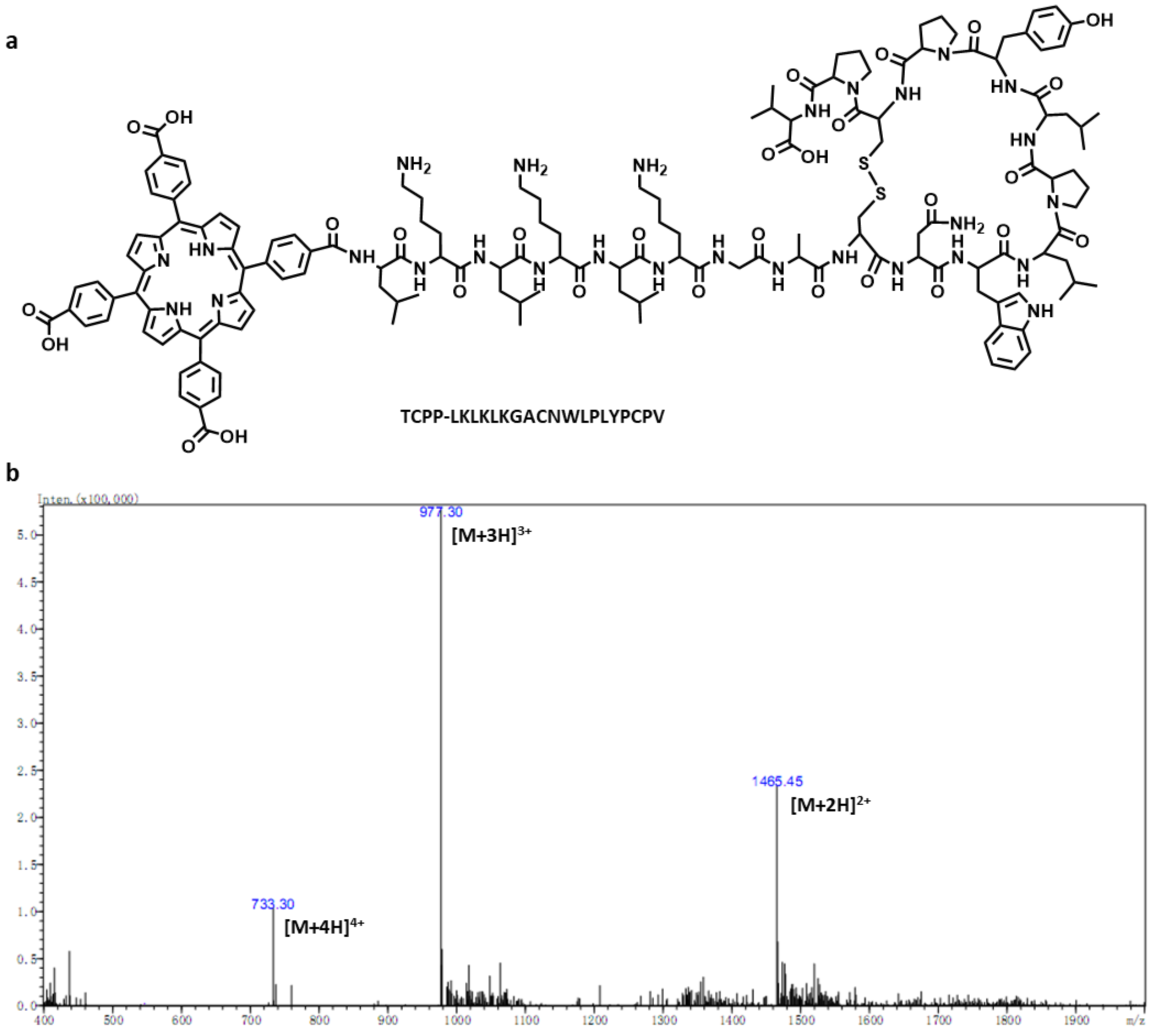

Supplementary Fig. 1. Molecular structure (**a**) and corresponding MALDI-TOF-MS spectrum (**b**) of TCPP-LKLKLKGACNWLPLYPCPV. Calculated molecular weight of TCPP-LKLKLKGACNWLPLYPCPV is 2927.

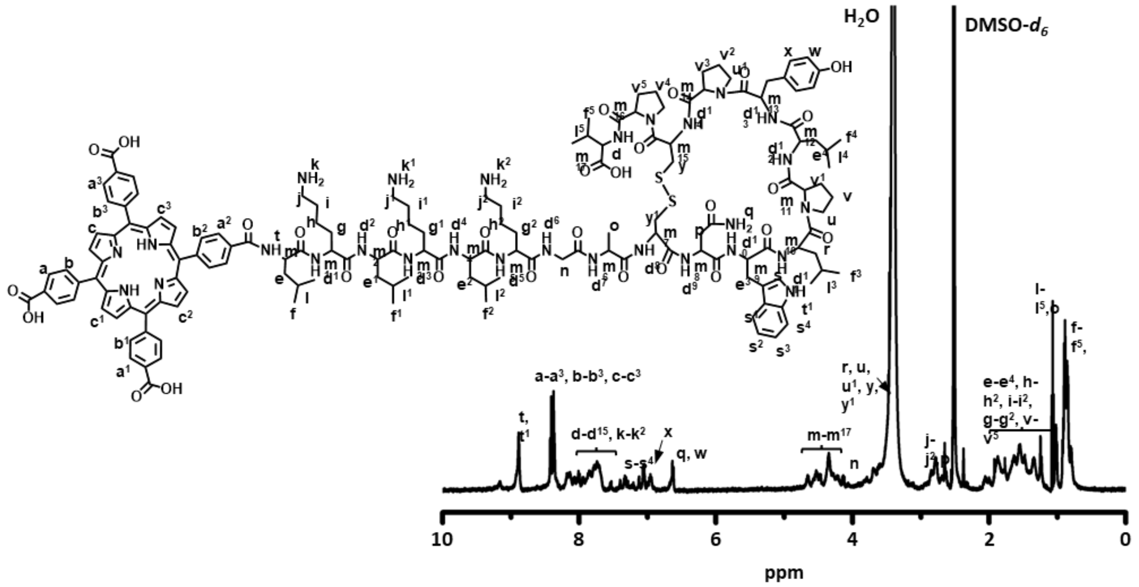

Supplementary Fig. 2. ^{1}H NMR (400 MHz, DMSO-d_6) spectrum of TCPP-LKLKLKGACNWLPLYPCPV.

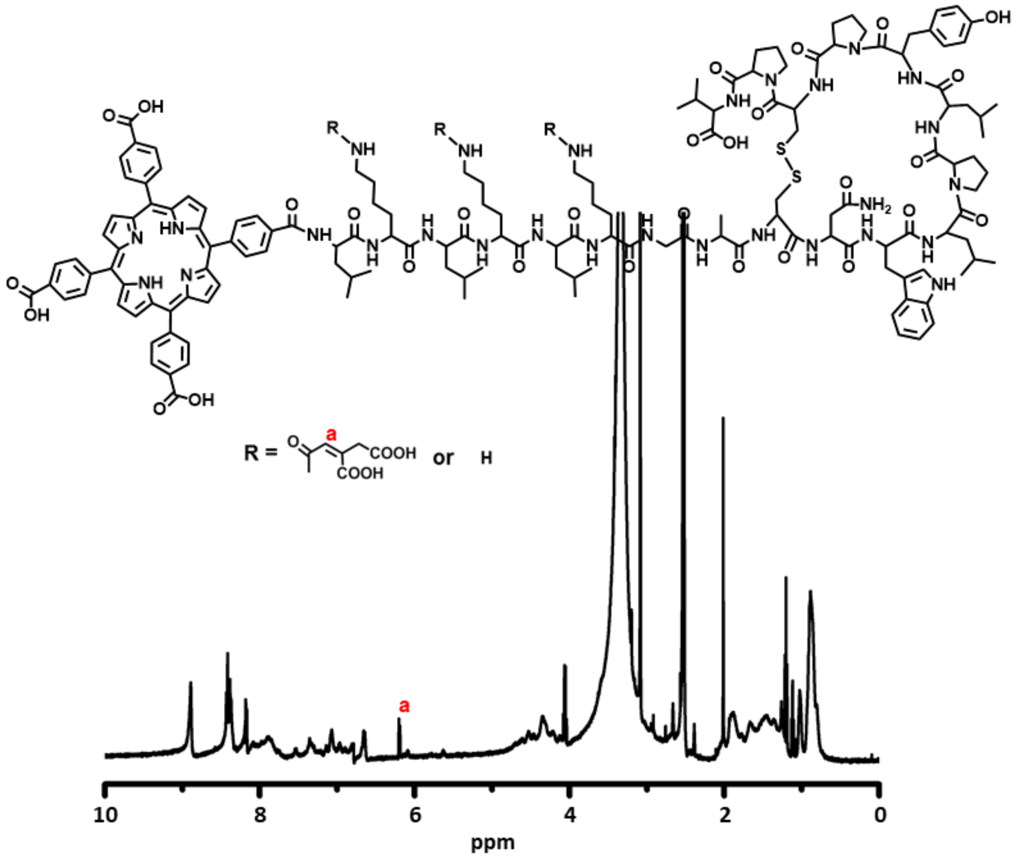

Supplementary Fig. 3. ^{1}H NMR (400 MHz, DMSO-d_6) spectrum of TCPP-LKLKLK(CAA)GACNWLPLYPCPV.

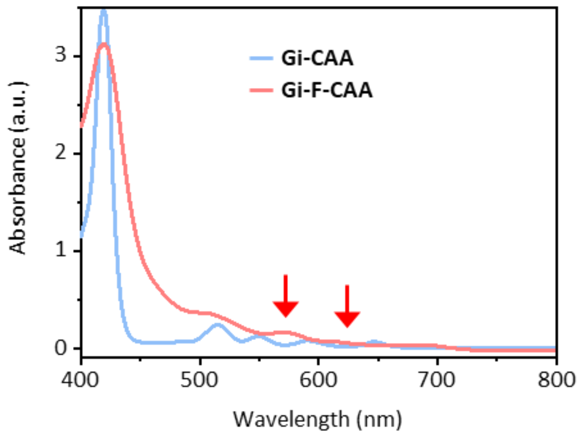

Supplementary Fig. 4. UV-visible absorption of Gi-CAA and **Gi-F-CAA**. The two characteristic Q-band peaks (red arrows) demonstrated the iron chelate.

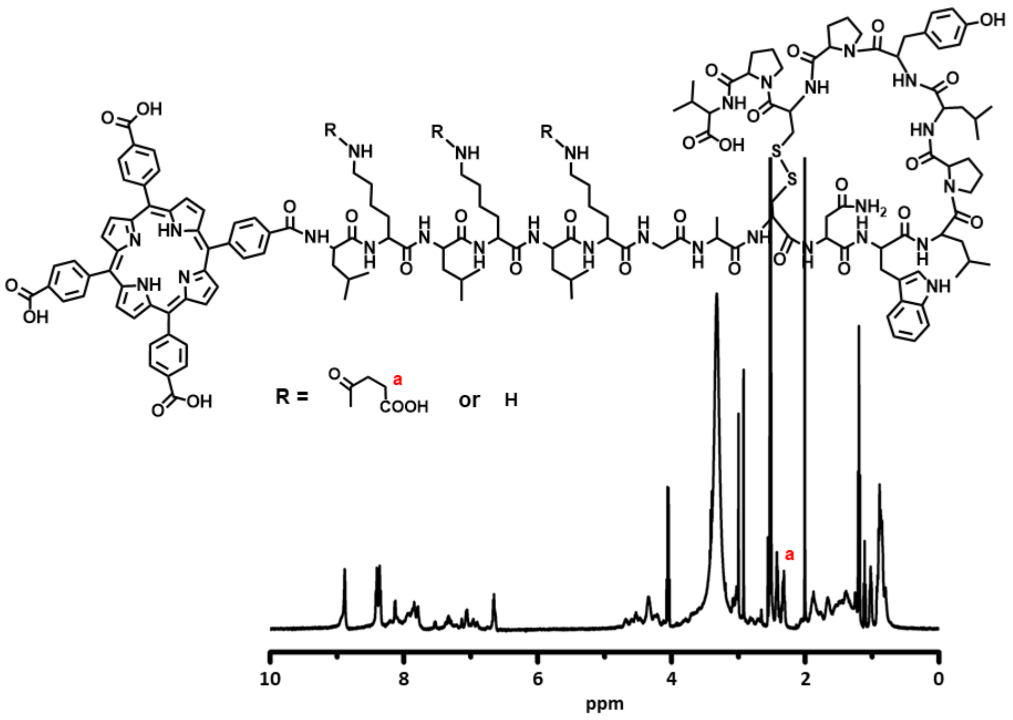

Supplementary Fig. 5. ^{1}H NMR (400 MHz, DMSO-d_6) spectrum of TCPP-LKLKLK(SA)GACNWLPLYPCPV.

a

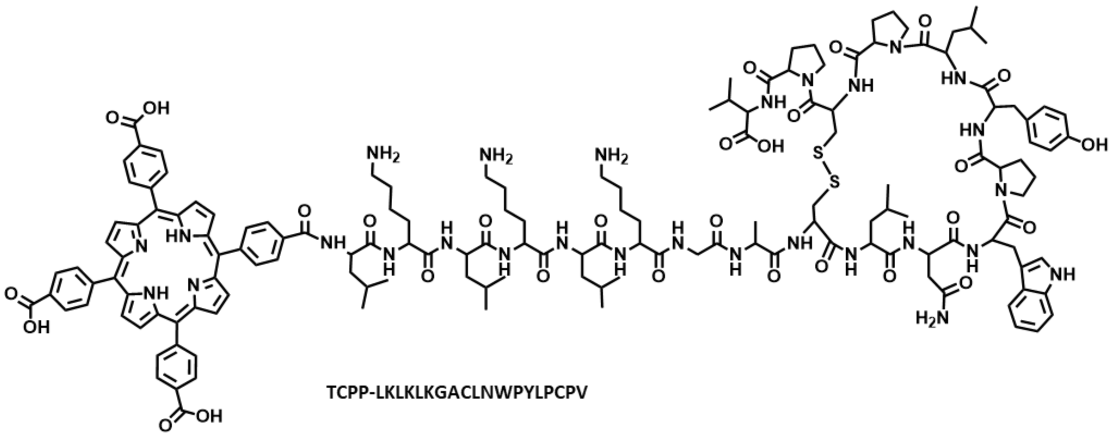

b

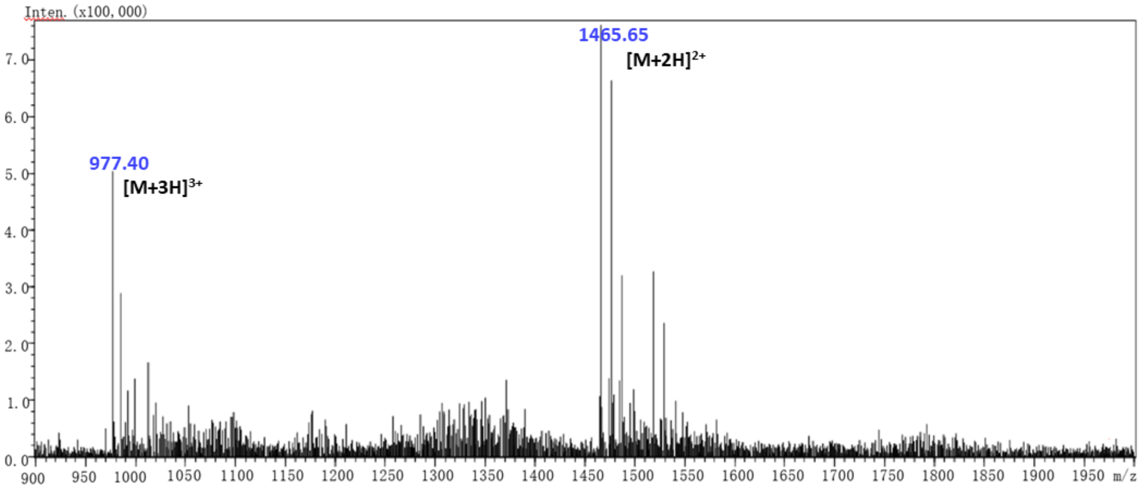

Supplementary Fig. 6. Molecular structure (**a**) and corresponding MALDI-TOF-MS spectrum (**b**) of TCPP-LKLKLKGACLNWPYLPCPV. Calculated molecular weight of TCPP-LKLKLKGACLNWPYLPCPV is 2927.

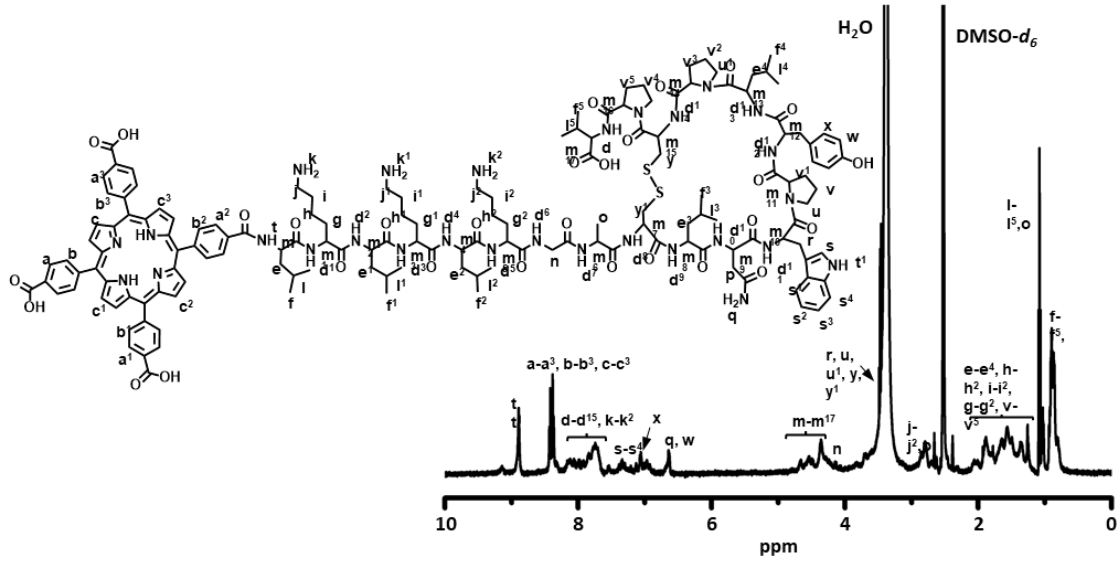

Supplementary Fig. 7. ^{1}H NMR (400 MHz, DMSO-d_6) spectrum of TCPP-LKLKLKGACLNWPYLPCPV.

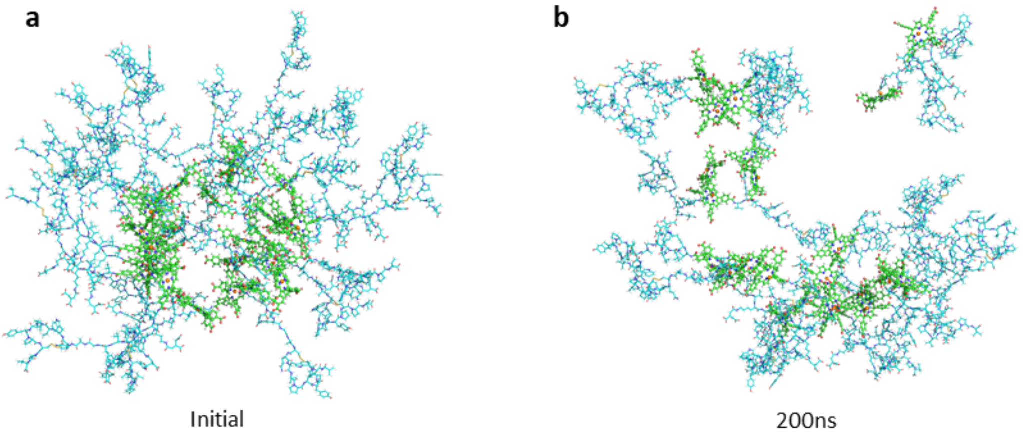

Supplementary Fig. 8. (**a**) The MD simulations of **Gi-F-CAA** begun from a beforehand nanosphere system. (**b**) The aggregations of **Gi-F-CAA**. The porphyrin parts were shown in sphere-stick mode with the carbon atoms colored in green and the other parts were shown in stick mode with the carbon atoms colored in cyan.

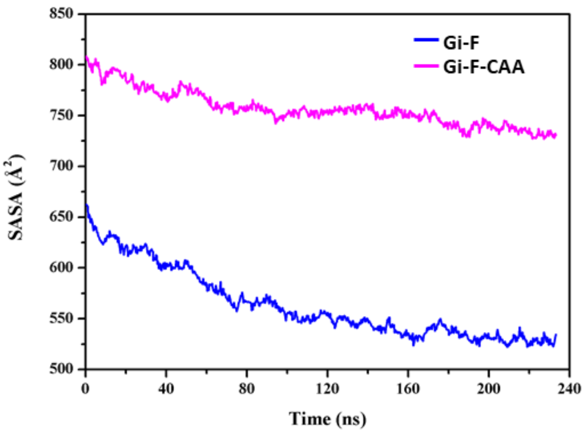

Supplementary Fig. 9. SASA values of Gi-F system and **Gi-F-CAA** system during MD simulations.

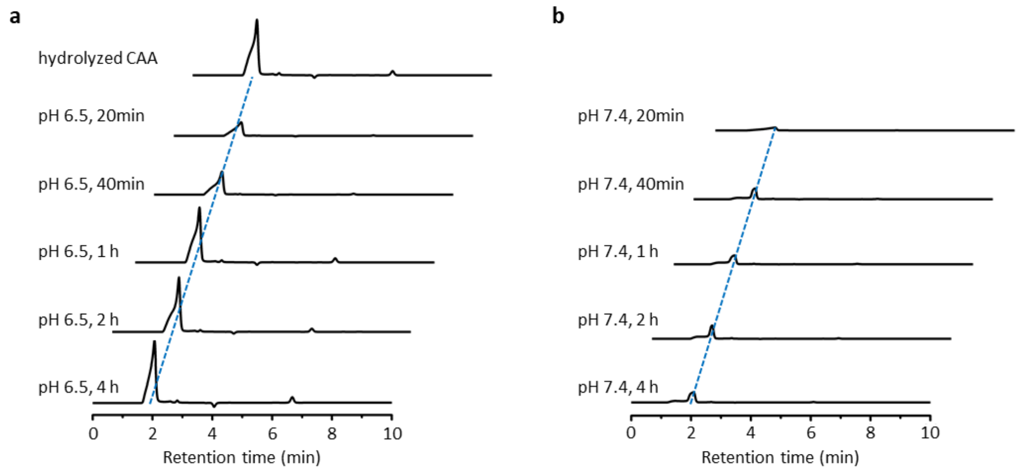

Supplementary Fig. 10. The HPLC chromatogram of the **Gi-F-CAA** (40 μM) dialyzed (MWCO: 1000) against (**a**) pH 6.5, (**b**) pH 7.4 buffer solutions at fixed time intervals.

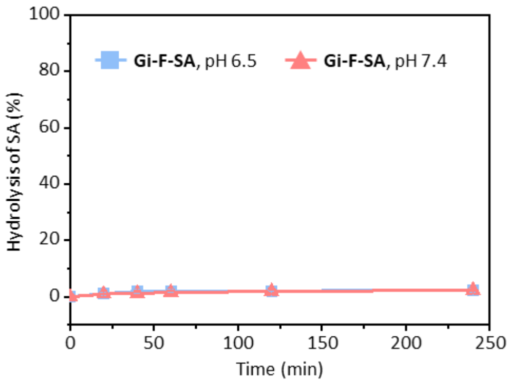

Supplementary Fig. 11. The hydrolysis profiles of Gi-F-SA (40 μM) at pH 7.4 and 6.5 (PBS, 0.01 M) measured by HPLC (n=3 experimental repeats). Experiment was independently repeated three times with similar results.

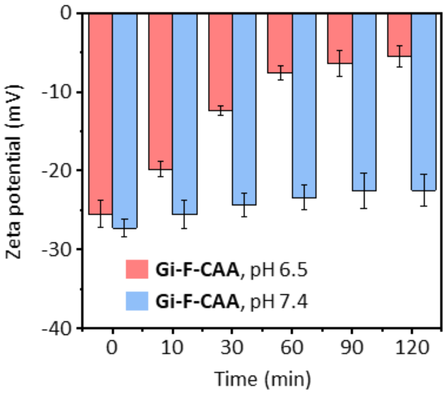

Supplementary Fig. 12. ζ-potential change of **Gi-F-CAA** (40 μM) in PBS (0.01 M, pH 7.4 or 6.5) (n=3 experimental repeats). Experiment was independently repeated three times with similar results. Values are expressed as means ± SD.

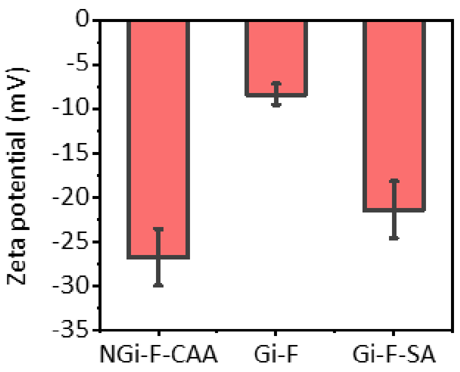

Supplementary Fig. 13. ζ-potential of NGi-F-CAA, Gi-F and Gi-F-SA (pH 7.4) (40 μM) in PBS (n=3 experimental repeats). Experiment was independently repeated three times with similar results. Values are expressed as means ± SD.

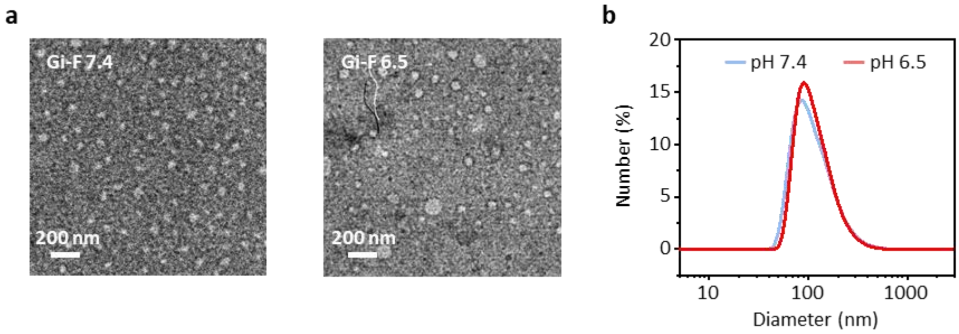

Supplementary Fig. 14. (a) Representative TEM images of Gi-F at pH 7.4 and 6.5 (40 μM). Scale bars: 200 nm. (b) Particle size of Gi-F at pH 7.4and 6.5) (40 μM) measured by DLS.

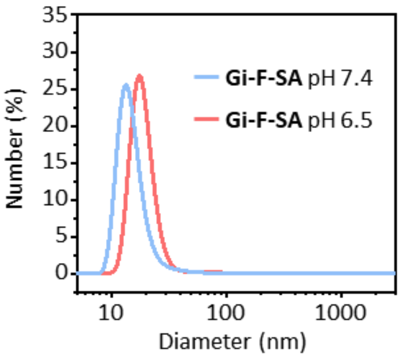

Supplementary Fig. 15. Particle size of Gi-F-SA at pH 7.4 and 6.5 (40 μM) measured by DLS.

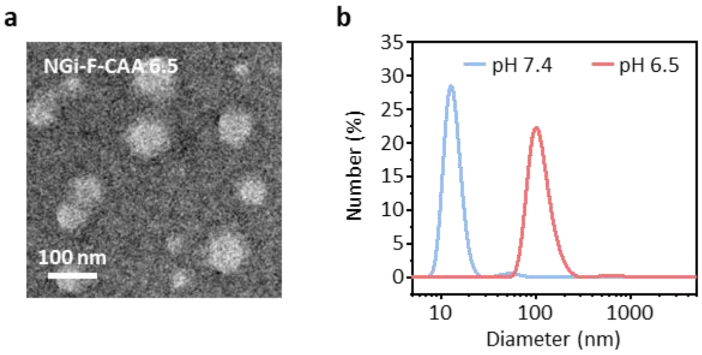

Supplementary Fig. 16. (**a**) Representative TEM images of NGi-F-CAA (pH 6.5) (40 μM). Scale bars: 100 nm. (**b**) Particle size of NGi-F-CAA at pH 7.4 and 6.5 (40 μM) measured by DLS.

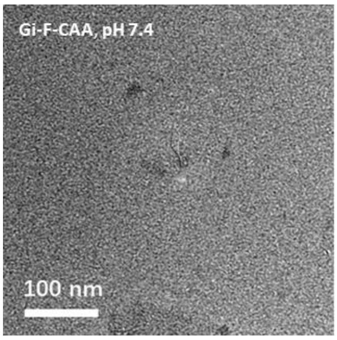

Supplementary Fig. 17. Representative TEM image of **Gi-F-CAA** (40 μM) in PBS at pH 7.4 for 1 h.

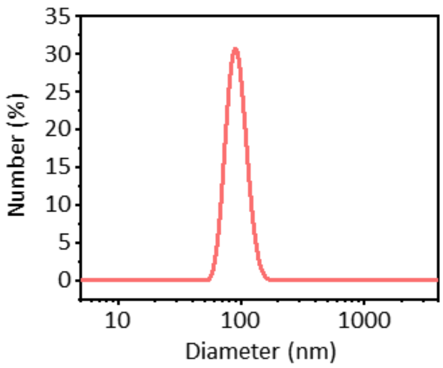

Supplementary Fig. 18. Particle size of Gi-F-CAA (40 μM) binding to GPX4 in PBS (0.01 M, pH 6.5) measured by DLS.

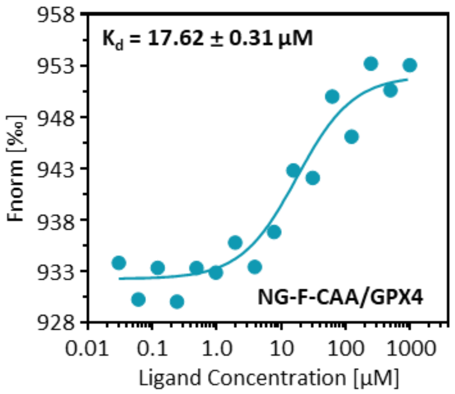

Supplementary Fig. 19. The binding affinity of **Gi-F-CAA** to GPX4 by Microscale Thermophoresis (MST) ligand binding measurements. K_d: the dissociation constant.

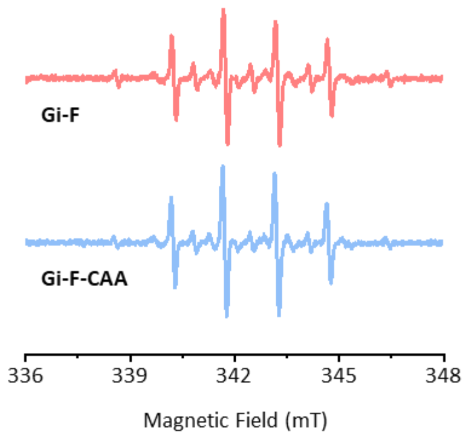

Supplementary Fig. 20. EPR spectra of DMPO/•OH adducts with **Gi-F-CAA** and Gi-F (40 μM) after treated with H_2O_2 (200 μM).

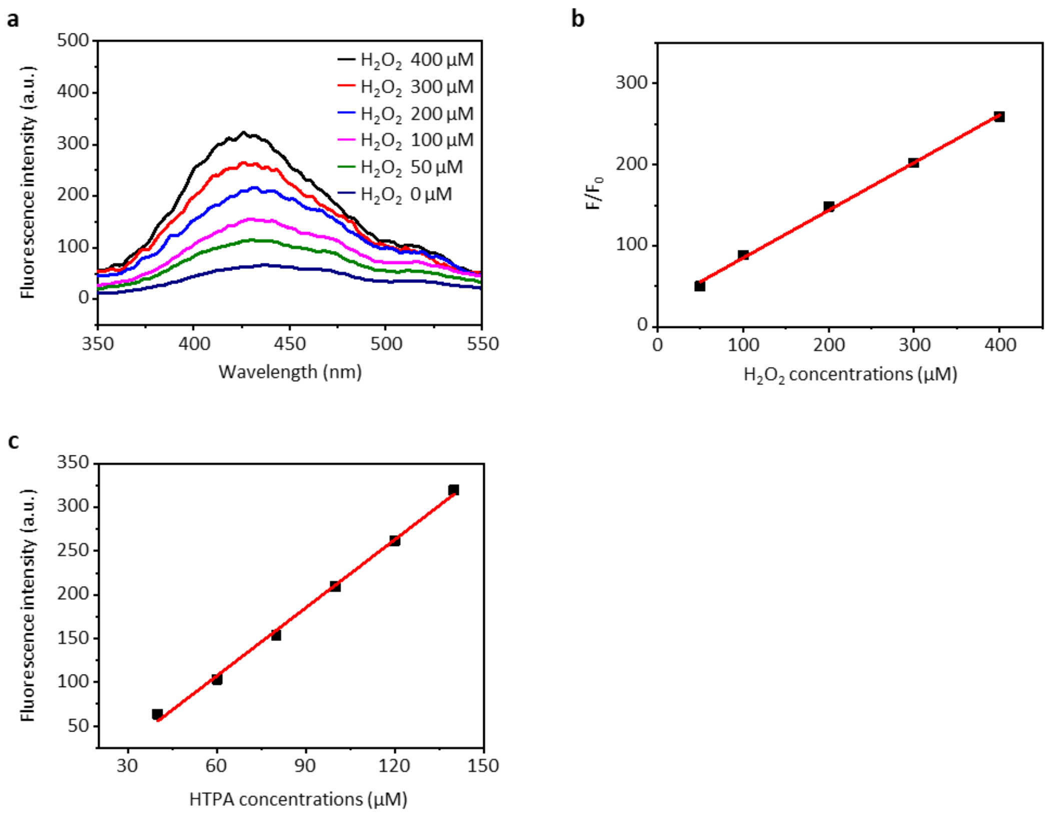

Supplementary Fig. 21. Terephthalic acid (TPA) reacts with hydroxyl radical in solution to produce stable and strong fluorescent substances (Ortho-hydroxyterephthalic acid, HTPA). The reaction is used to quantitatively analyze •OH produced by Gi-F under the H_2O_2 treatment (**a**). Plot showing the linear relationship of relative fluorescence intensity with H_2O_2 concentration (**b**). The fluorescence of different concentration of HTPA at 425 nm (λex = 310 nm) (**c**).

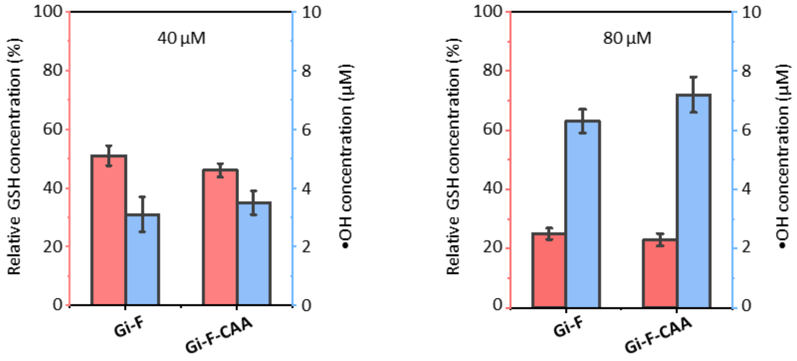

Supplementary Fig. 22. Relative GSH concentration depletion and •OH generation of Gi-F-CAA and Gi-F (40 μM or 80 μM) after incubation with H_2O_2 (200 μM) (n=3 experimental repeats). Experiment was independently repeated three times with similar results.

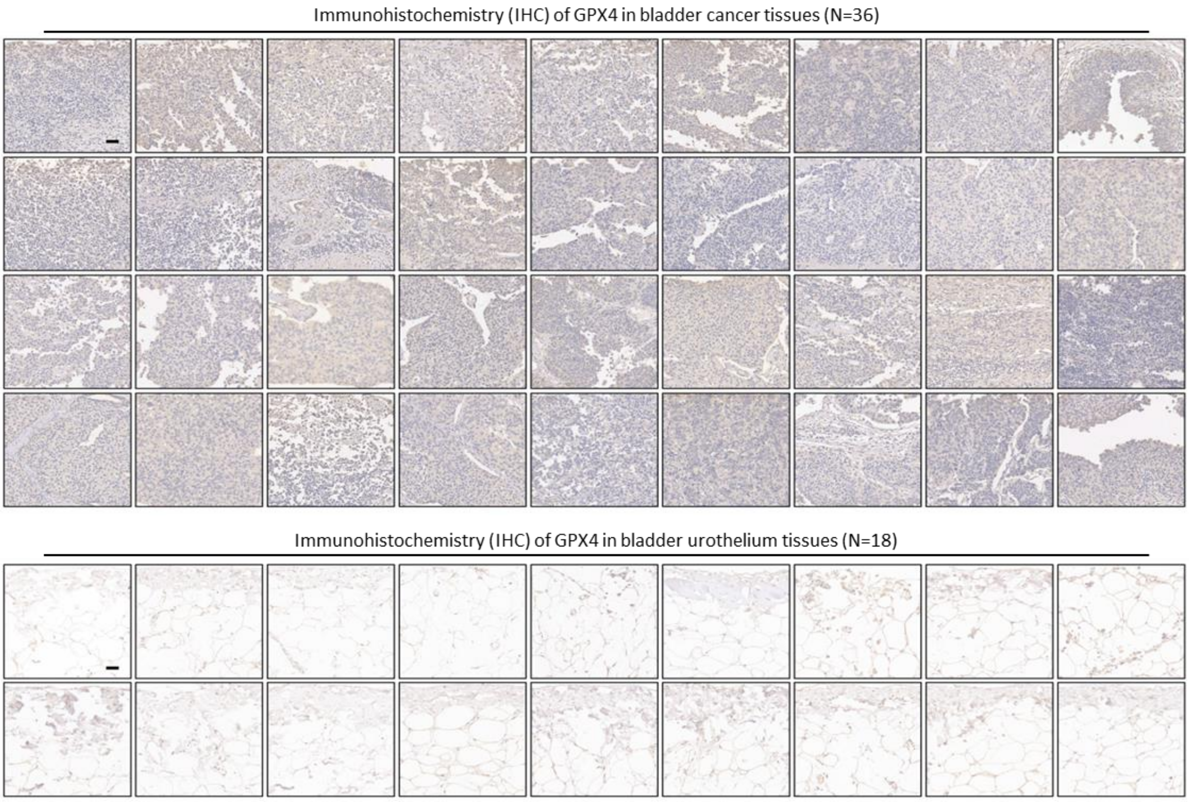

Supplementary Fig. 23. GPX4 immunohistochemical staining of bladder tumor tissues and normal bladder tissues. Scale bars: 50 μm.

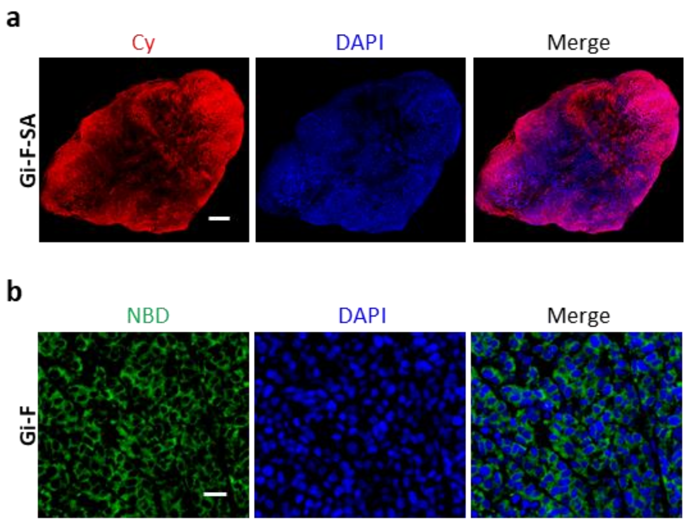

Supplementary Fig. 24. (**a**) Fluorescence of images of the whole tumor tissues after treated with Cy labeled Gi-F-SA (8 mg/kg in 100 μL PBS). Scale bars: 1 mm. (**b**) Fluorescence images of tumor tissues after treated with NBD labeled Gi-F (8 mg/kg in 100 μL PBS). Scale bars: 20 μm.

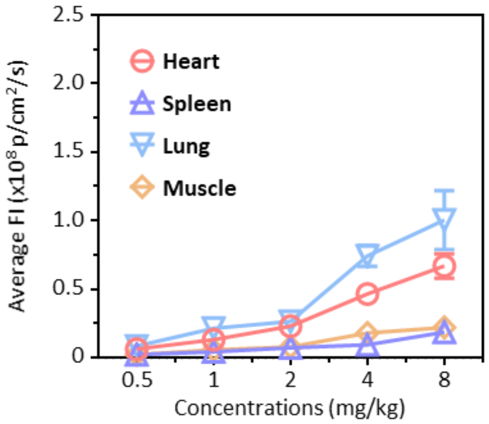

Supplementary Fig. 25. Quantitative statistical analysis for the biodistribution of **Gi-F-CAA** in heart, spleen, lung and muscle (n = 3 mice).

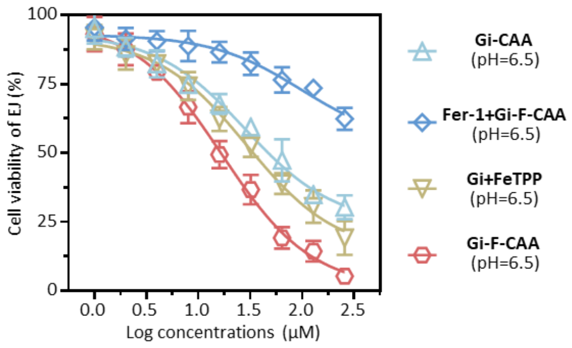

Supplementary Fig. 26. The viability of EJ cells after treated with Gi-CAA (pH=6.5), Fer-1+**Gi-F-CAA** (pH=6.5) and **Gi-F-CAA** (pH=6.5) at different concentrations for 48 h (n=3 experimental repeats). Experiment was independently repeated three times with similar results.

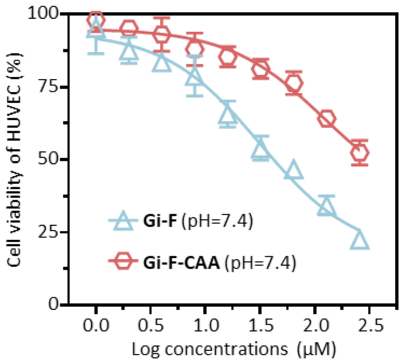

Supplementary Fig. 27. Viability of HUVEC cells after treated with Gi-F (pH=7.4) and **Gi-F-CAA** (pH=7.4) at different concentrations for 48 h (n=3 experimental repeats). Experiment was independently repeated three times with similar results.

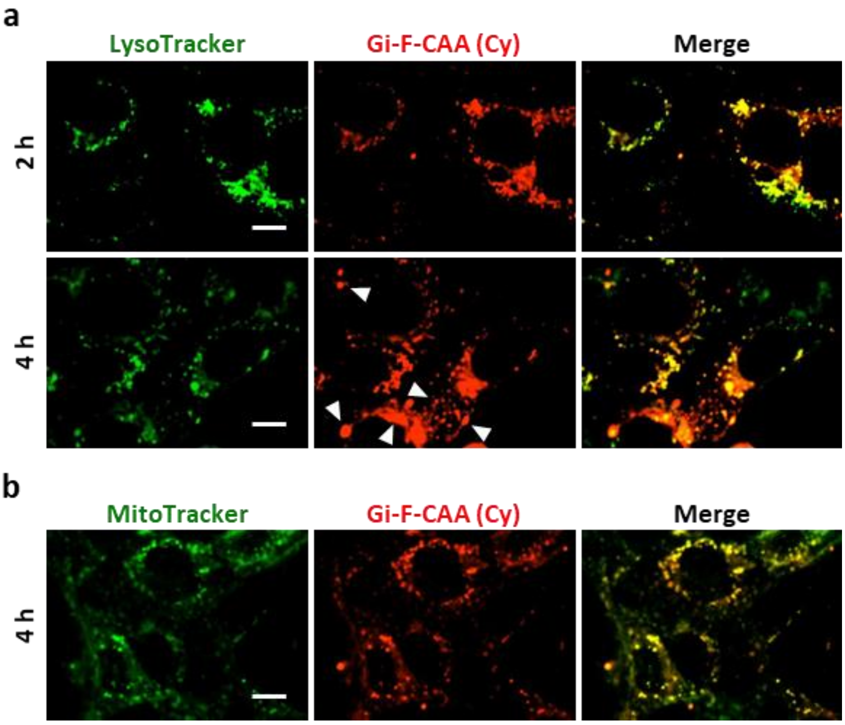

Supplementary Fig. 28. (**a**) Representative fluorescence images of EJ cells after incubation with Cy labeled **Gi-F-CAA** (pH 6.5, 40 μM) for 2 and 4 h. Lysosomes were labeled with LysoTracker Green. Scale bar: 10 μm. (**b**) Representative fluorescence images of EJ cells after incubation with Cy labeled **Gi-F-CAA** (pH 6.5, 40 μM) for 4 h. Mitochondria were labeled with MitoTracker Green. Scale bar: 10 μm.

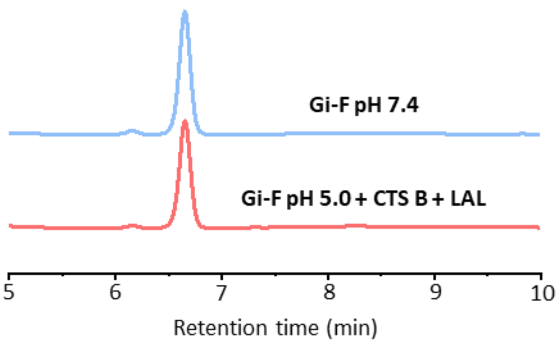

Supplementary Fig. 29. HPLC spectra of Gi-F (pH 7.4, 40 μM) and Gi-F + CTS + LAL (pH 5.0, 40 μM). CTS: Cathepsin. LAL: lysosomalacidllpase.

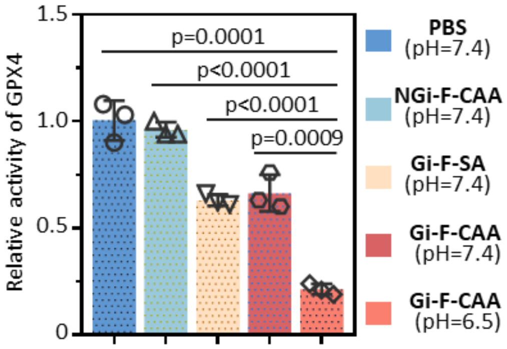

Supplementary Fig. 30. Glutathione peroxidase 4 (GPX4) activities after treated with PBS (pH=7.4), NGi-F-CAA (pH=7.4), Gi-F-SA (pH=7.4), **Gi-F-CAA** (pH=7.4) and **Gi-F-CAA** (pH=6.5, 40 μM) (n=3 experimental repeats). Experiment was independently repeated three times with similar results. The GPX4 activities of PBS group was normalized as 1. p value was performed with one-way ANOVA followed by post hoc Tukey's test. Data were presented as mean ± SD.

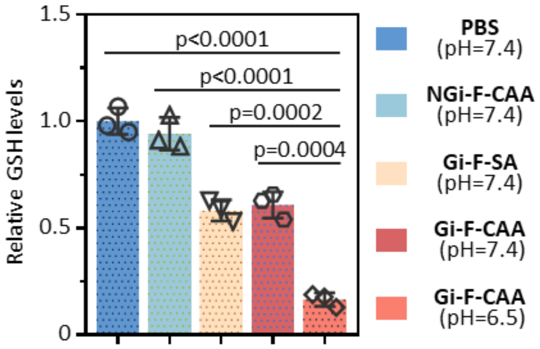

Supplementary Fig. 31. Intracellular GSH levels of EJ cells after treated with PBS (pH=7.4), NGi-F-CAA (pH=7.4), Gi-F-SA (pH=7.4), **Gi-F-CAA** (pH=7.4) and **Gi-F-CAA** (pH=6.5, 40 μM) (n=3 experimental repeats). Experiment was independently repeated three times with similar results. The GSH levels of PBS group was normalized as 1. p value was performed with one-way ANOVA followed by post hoc Tukey's test. Data were presented as mean ± SD.

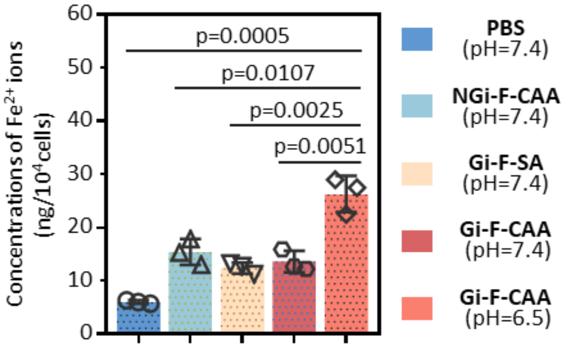

Supplementary Fig. 32. Intracellular Fe^{2+} ions activities of EJ cells after treated with PBS (pH=7.4), NGi-F-CAA (pH=7.4), Gi-F-SA (pH=7.4), **Gi-F-CAA** (pH=7.4) and **Gi-F-CAA** (pH=6.5, 40 μM) (n=3 experimental repeats). Experiment was independently repeated three times with similar results. p value was performed with one-way ANOVA followed by post hoc Tukey's test. Data were presented as mean ± SD.

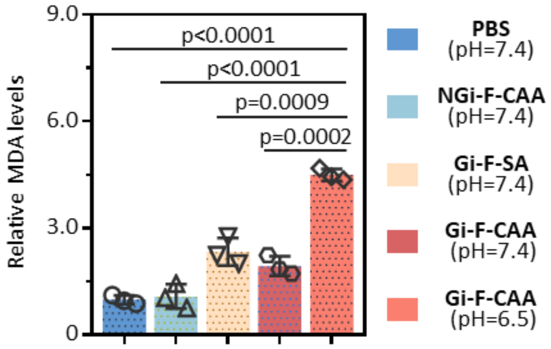

Supplementary Fig. 33. Intracellular malonaldehyde (MDA) levels of EJ cells after treated with PBS (pH=7.4), NGi-F-CAA (pH=7.4), Gi-F-SA (pH=7.4), **Gi-F-CAA** (pH=7.4) and **Gi-F-CAA** (pH=6.5, 40 μM) (n=3 experimental repeats). Experiment was independently repeated three times with similar results. The MDA levels of PBS group was normalized as 1. p value was performed with one-way ANOVA followed by post hoc Tukey's test. Data were presented as mean ± SD.

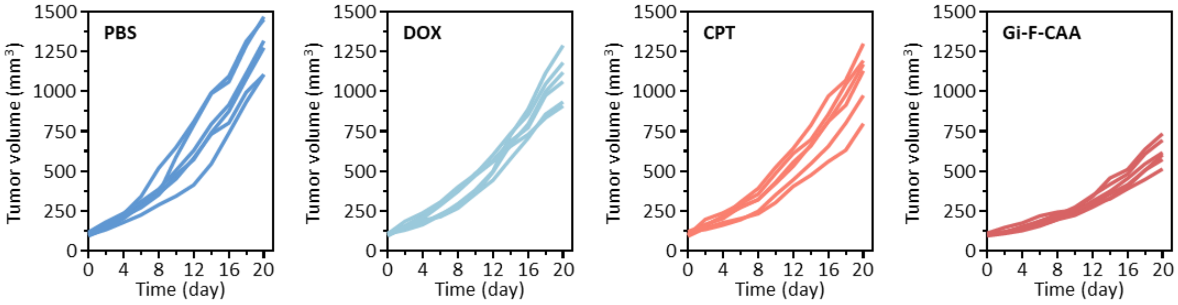

Supplementary Fig. 34. The individual tumor growth curves of mice after treated with PBS, DOX (3 mg/kg in 100 μL PBS), CPT (3 mg/kg in 100 μL PBS) and **Gi-F-CAA** (8 mg/kg in 100 μL PBS) over 20 days.

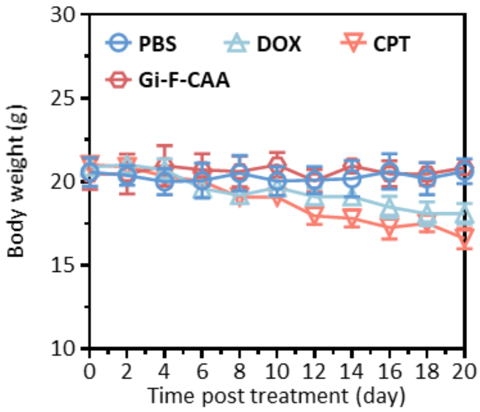

Supplementary Fig. 35. Body weight changes of MCF-7/MDR xenografted mice after treated with PBS, DOX (3 mg/kg in 100 μL PBS), CPT (3 mg/kg in 100 μL PBS) and **Gi-F-CAA** (8 mg/kg in 100 μL PBS) over 20 days (n=6 mice). Data were presented as mean ± SD.

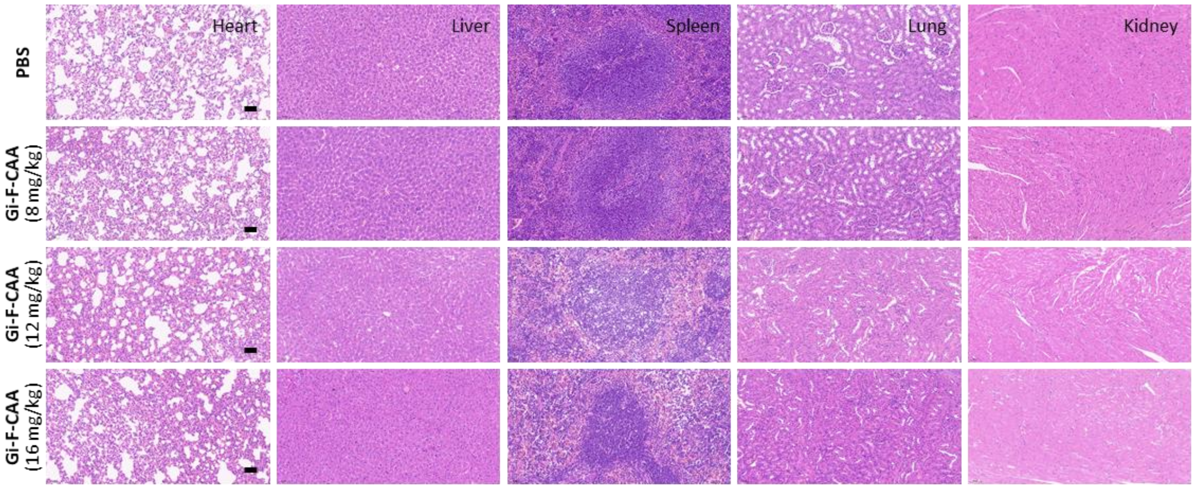

Supplementary Fig. 36. Histology evaluation of the major organs by hematoxylin and eosin (H&E) after treatment with PBS and **Gi-F-CAA** (8, 12 and 16 mg/kg in 100 μL PBS) for 1 days (n = 4 mice). Scale bar: 50 μm.

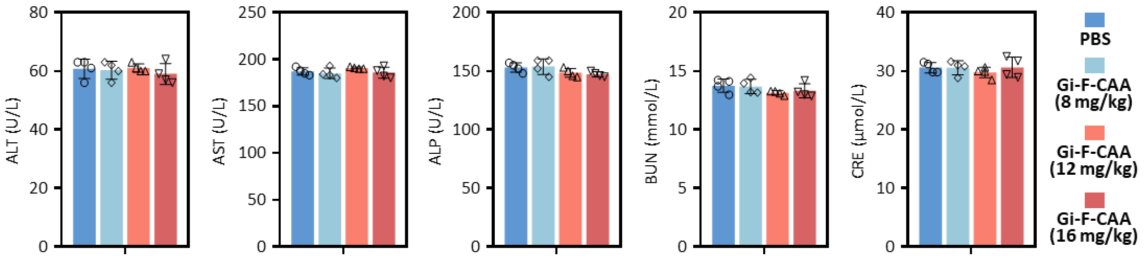

Supplementary Fig. 37. Blood biochemistry data of the mice including alanine aminotransferase (ALT), aspartate aminotransferase (AST), alkaline phosphatase (ALP), blood urea nitrogen (BUN) and creatinine (CRE) after treatment with PBS and **Gi-F-CAA** (8, 12 and 16 mg/kg in 100 μL PBS) for 1 days (n=4 mice). Data were presented as mean ± SD.

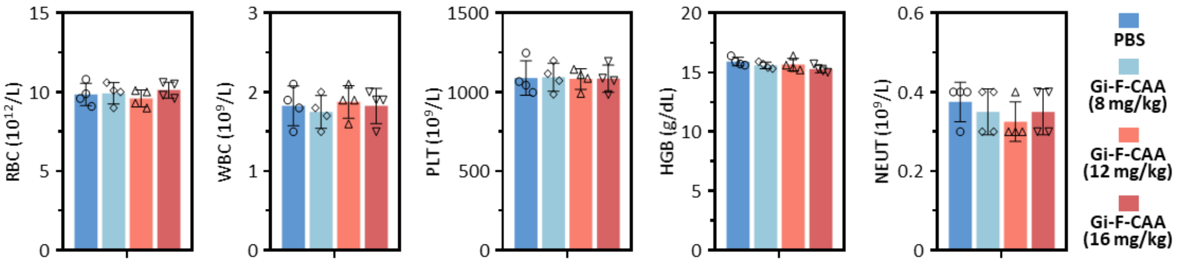

Supplementary Fig. 38. Blood routine examination of the mice including red blood cell (RBC), white blood cell (WBC), platelets (PLT), haemoglobin (HGB) and neutrophil (NEUT) after treatment with PBS and **Gi-F-CAA** (8, 12 and 16 mg/kg in 100 μL PBS) for 1 days (n=4 mice). Data were presented as mean ± SD.
