# Supplementary material for: In vivo assembly enhanced binding effect augments tumor specific ferroptosis therapy
Source: Nat Commun. 2024 Jan 11;15:454. doi: 10.1038/s41467-023-44665-2 (PMC10784468; doi:10.1038/s41467-023-44665-2)
Supplement: Supplementary file 3 — Reporting Summary [file 41467_2023_44665_MOESM3_ESM.pdf]

nature portfolio

Corresponding author(s): Hao Wang

Last updated by author(s): Nov 20, 2023

Reporting Summary

Nature Portfolio wishes to improve the reproducibility of the work that we publish. This form provides structure for consistency and transparency in reporting. For further information on Nature Portfolio policies, see our Editorial Policies and the Editorial Policy Checklist.

Statistics

For all statistical analyses, confirm that the following items are present in the figure legend, table legend, main text, or Methods section.

n/a	Confirmed	
☐	☒	The exact sample size (n) for each experimental group/condition, given as a discrete number and unit of measurement
☐	☒	A statement on whether measurements were taken from distinct samples or whether the same sample was measured repeatedly
☐	☒	The statistical test(s) used AND whether they are one- or two-sided<br>*Only common tests should be described solely by name; describe more complex techniques in the Methods section.*
☒	☐	A description of all covariates tested
☒	☐	A description of any assumptions or corrections, such as tests of normality and adjustment for multiple comparisons
☐	☒	A full description of the statistical parameters including central tendency (e.g. means) or other basic estimates (e.g. regression coefficient) AND variation (e.g. standard deviation) or associated estimates of uncertainty (e.g. confidence intervals)
☐	☒	For null hypothesis testing, the test statistic (e.g. F, t, r) with confidence intervals, effect sizes, degrees of freedom and P value noted<br>*Give P values as exact values whenever suitable.*
☒	☐	For Bayesian analysis, information on the choice of priors and Markov chain Monte Carlo settings
☒	☐	For hierarchical and complex designs, identification of the appropriate level for tests and full reporting of outcomes
☐	☒	Estimates of effect sizes (e.g. Cohen's d, Pearson's r), indicating how they were calculated

Our web collection on statistics for biologists contains articles on many of the points above.

Software and code

Policy information about availability of computer code

Data collection	Transmission Electron Microscope (HT7700); Dynamic Light Scattering (Nano-ZS 3600, Malvern Instruments, UK); Confocal laser scanning microscopy (UltraVIEW Vox); in vivo imaging system (IVIS Spectrum); blood biochemistry analysis (Hitachi Automatic Biochemical Analyzer 7100); blood routine analysis and histology evaluation (Servicebio Technology Co., Ltd.)
Data analysis	Bar graph, curves and the relevant statistics were analyzed by GraphPad Prism 8.0 and origin 9.0; Confocal data were analyzed by VolocityDemo; Molecular dynamics simulations data were analyzed by Molecular Operating Environment (MOE2020) and Packmol.

For manuscripts utilizing custom algorithms or software that are central to the research but not yet described in published literature, software must be made available to editors and reviewers. We strongly encourage code deposition in a community repository (e.g. GitHub). See the Nature Portfolio guidelines for submitting code & software for further information.

Data

Policy information about availability of data

All manuscripts must include a data availability statement. This statement should provide the following information, where applicable:

- Accession codes, unique identifiers, or web links for publicly available datasets
- A description of any restrictions on data availability
- For clinical datasets or third party data, please ensure that the statement adheres to our policy

The data supporting the findings of this study are available within the article and Supplementary Information files. Source data are provided with this paper. Correspondence and requests for materials should be addressed to T.-L. Sun, Z.-Y. Qiao, W.-H. Xu and H. Wang (wanghao@nanoctr.cn).

Research involving human participants, their data, or biological material

Policy information about studies with human participants or human data. See also policy information about sex, gender (identity/presentation), and sexual orientation and race, ethnicity and racism.

Reporting on sex and gender	Sex and gender of bladder cancer patient has been collected and recorded by authors, which has no bearing on data analysis or results.
Reporting on race, ethnicity, or other socially relevant groupings	Not collected, therefore not reported. No relevance to data.
Population characteristics	Age of bladder cancer patient has been collected and recorded by authors.
Recruitment	Bladder cancer patients were randomly recruited to collect bladder tumor tissues and normal bladder tissues. Inclusion criteria: (1) Bladder cancer patients who have signed the informed consent form before any study-related procedures. Patients have fully understood the study and voluntarily signed the informed consent form in writing. (2) Intended for surgical treatment. (3) Aged between 18 and 70. (4) Karnofsky Performance Status (KPS) score ≥60. (5) Basic normal organ function; neutrophil count >1.5×109/L, platelet count >100×109/L, hemoglobin >9.0 g/dL. Total bilirubin is normal or <1.5× ULN; AST (SGOT) and ALT (SGPT) <2.5×ULN (if liver metastasis is present, <5×ULN); serum creatinine is <1.5×ULN. 6. Expected survival period ≥3 months. Exclusion criteria: (1) Patients with abnormalities in the urinary system or urethral stricture. (2) Pregnant or lactating women. (3) with severe concomitant diseases in other systems. (4) Patients allergic to multiple drugs. (5) Patients who are currently participating or have participated in another clinical study within 30 days. None of the above inclusion criteria or exclusion criteria affected the experimental results.
Ethics oversight	All the experiments performed with human specimens were reviewed and approved by the Committees for Ethical Review of the Fourth Hospital of Harbin Medical University (2022-SCILLSC-28)

Note that full information on the approval of the study protocol must also be provided in the manuscript.

Field-specific reporting

Please select the one below that is the best fit for your research. If you are not sure, read the appropriate sections before making your selection.

☒ Life sciences ☐ Behavioural & social sciences ☐ Ecological, evolutionary & environmental sciences

For a reference copy of the document with all sections, see nature.com/documents/nr-reporting-summary-flat.pdf

Life sciences study design

All studies must disclose on these points even when the disclosure is negative.

Sample size	We choose six mice in each group for treatment, which is representative of treatment outcomes. Sample sizes were based on our previous experience and other publications (Nat Commun. 2022; 13: 418; Nat Commun. 2019; 10: 4861.), providing enough statistical power to detect the usually strong effects observed in our experiments. All sample sizes are clearly described in the manuscript or the figure legend.
Data exclusions	No data were excluded from the analyses.
Replication	All the experiments were performed in triplicates with a good reproducibility of the experimental findings.
Randomization	All samples including animals and cells were randomly allocated into different groups.
Blinding	The investigators were blinded to group allocation during data collection and/or analysis.

Reporting for specific materials, systems and methods

We require information from authors about some types of materials, experimental systems and methods used in many studies. Here, indicate whether each material, system or method listed is relevant to your study. If you are not sure if a list item applies to your research, read the appropriate section before selecting a response.

Materials & experimental systems

n/a	Involved in the study
☐	☒ Antibodies
☐	☒ Eukaryotic cell lines
☒	☐ Palaeontology and archaeology
☐	☒ Animals and other organisms
☒	☐ Clinical data
☒	☐ Dual use research of concern
☒	☐ Plants

Methods

n/a	Involved in the study
☒	☐ ChIP-seq
☒	☐ Flow cytometry
☒	☐ MRI-based neuroimaging

Antibodies

Antibodies used	Anti-GPX4 antibody: from Abcam, cat: ab125066;
Validation	Anti-GPX4 antibody suitable for: Flow Cyt (Intra), WB, IHC-P, ICC/IF;

Eukaryotic cell lines

Policy information about cell lines and Sex and Gender in Research

Cell line source(s)	EJ bladder cancer cell line, MCF-7/MDR breast cancer cell line and 786-O renal cell carcinoma cell line were purchased from the Cell Culture Center of the Institute of Basic Medicine, Chinese Academy of Medical Sciences (Beijing, China).
Authentication	None of the cell lines used were authenticated.
Mycoplasma contamination	The cell lines were not tested for mycoplasma contamination.
Commonly misidentified lines (See ICLAC register)	No commonly misidentified cell lines was used in this study.

Animals and other research organisms

Policy information about studies involving animals; ARRIVE guidelines recommended for reporting animal research, and Sex and Gender in Research

Laboratory animals	BALB/c nude mice (8 weeks old, 18 g) were purchased from Vital River Laboratory Animal Technology Co., Ltd. (Beijing, China).
Wild animals	The study did not involve wild animals.
Reporting on sex	Sex and gender of BALB/c nude mice has been collected and recorded by authors, which has no bearing on data analysisor results.
Field-collected samples	The study did not involve samples collected from the field.
Ethics oversight	All the animal experiments were conducted in compliance with ethical compliance and approval by the Institutional Animal Care and Use Committee of National Center for Nanoscience and Technology (NCNST21-2208-0608).

Note that full information on the approval of the study protocol must also be provided in the manuscript.
