# Supplementary material for: In vivo assembly enhanced binding effect augments tumor specific ferroptosis therapy
Source: Nat Commun. 2024 Jan 11;15:454. doi: 10.1038/s41467-023-44665-2 (PMC10784468; doi:10.1038/s41467-023-44665-2)
Supplement: Supplementary file 4 — Source Data [file 41467_2023_44665_MOESM4_ESM.zip › Source Data/Figure 1/Figure 1a/Figure 1a.pdf]

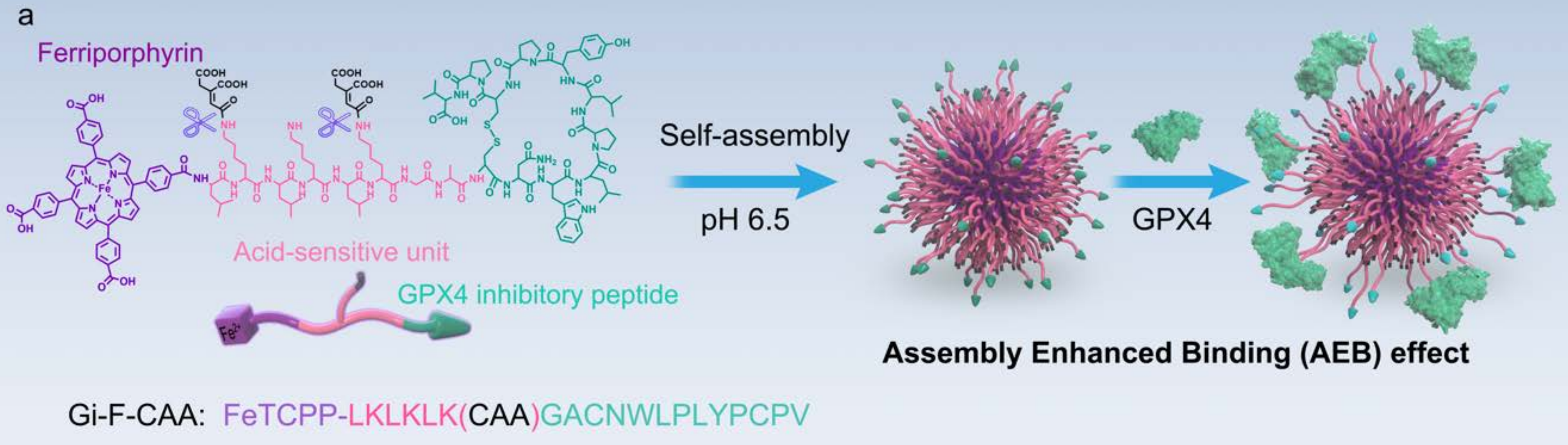
a
Ferriporphyrin
Acid-sensitive unit
GPX4 inhibitory peptide
Self-assembly
pH 6.5
GPX4
Assembly Enhanced Binding (AEB) effect
Gi-F-CAA: FeTCPP-LKLKLK(CAA)GACNWLPLYPCPV
