# Supplementary figures and images for: In vivo assembly enhanced binding effect augments tumor specific ferroptosis therapy
Source: Nat Commun. 2024 Jan 11;15:454. doi: 10.1038/s41467-023-44665-2 (PMC10784468; doi:10.1038/s41467-023-44665-2)

b

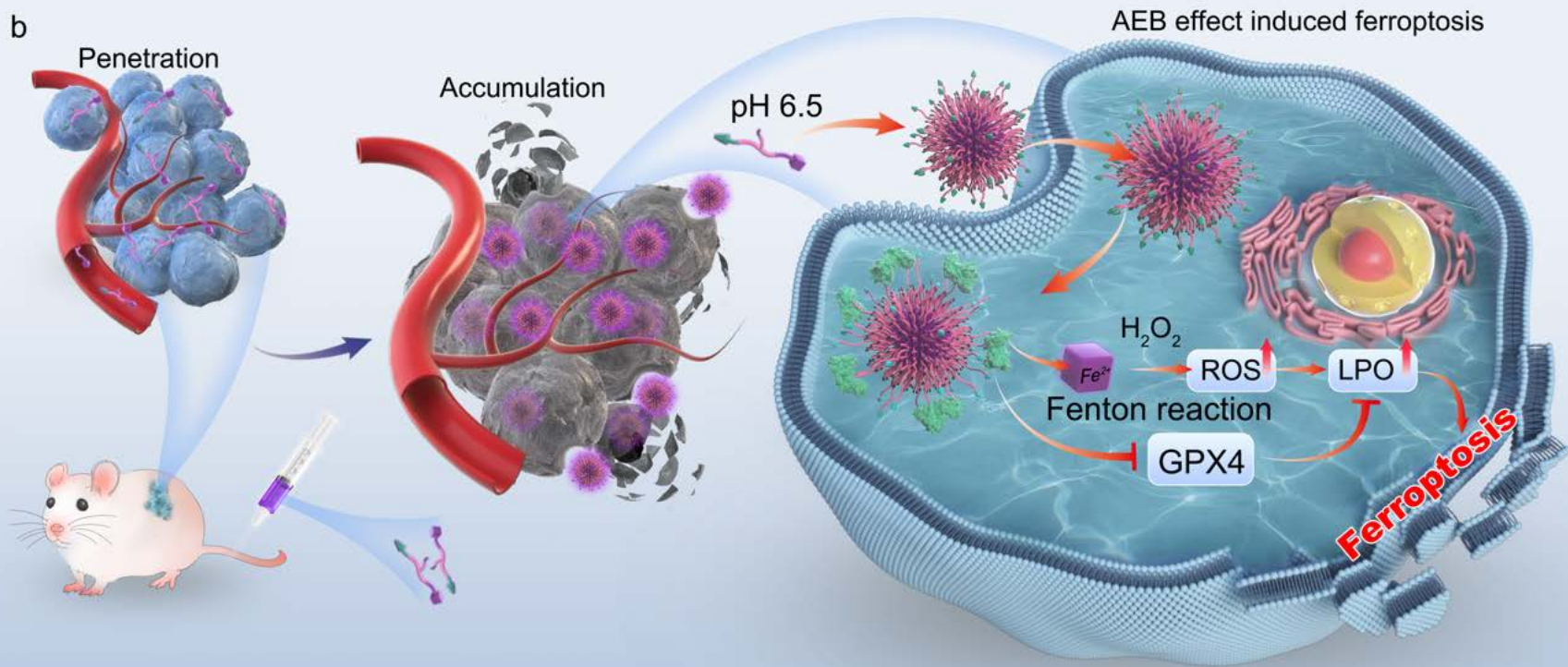

Supplement: Supplementary file 4 — Source Data [file 41467_2023_44665_MOESM4_ESM.zip › Source Data/Figure 1/Figure 1b/Figure 1b.pdf]

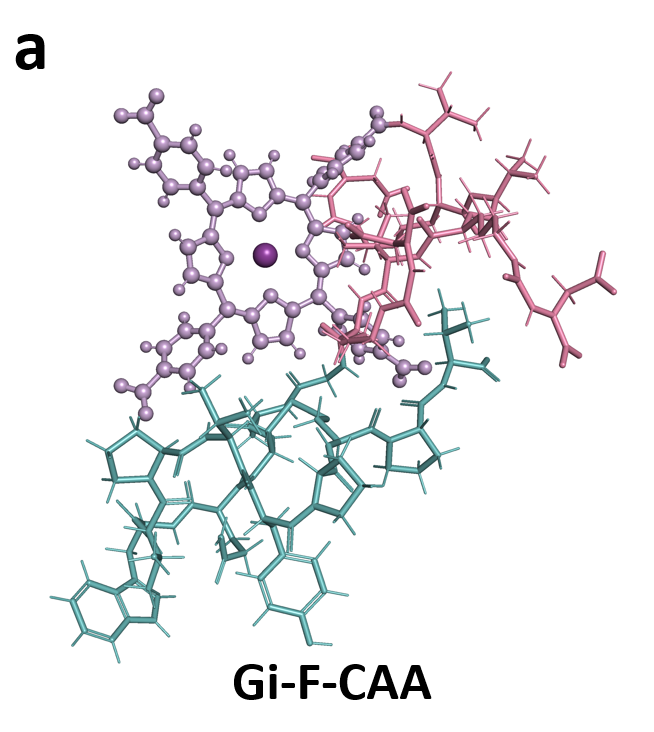

Supplement: Supplementary file 4 — Source Data [file 41467_2023_44665_MOESM4_ESM.zip › Source Data/Figure 2/Figure 2a/Figure 2a.png]

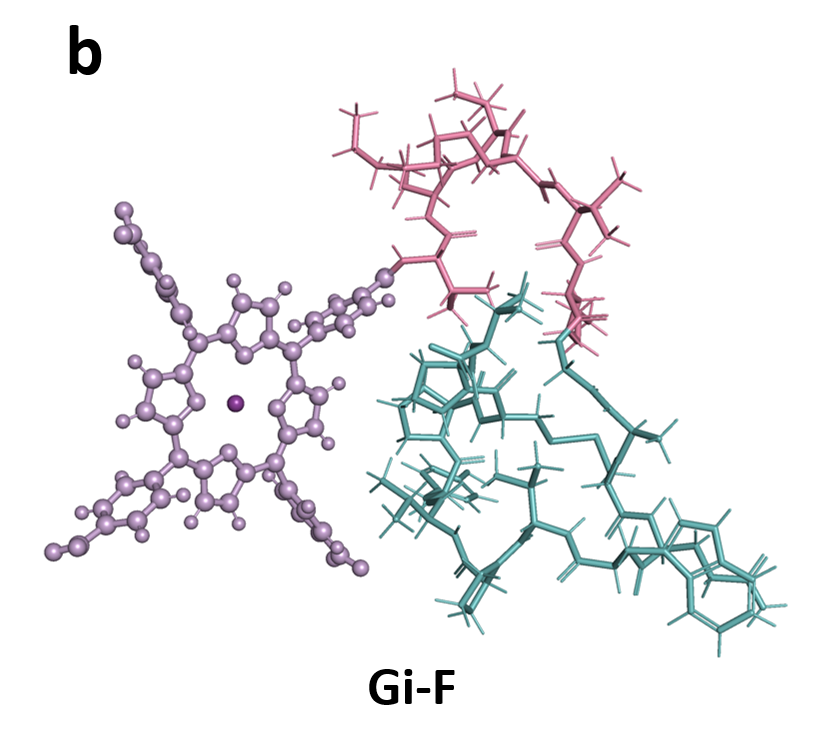

Supplement: Supplementary file 4 — Source Data [file 41467_2023_44665_MOESM4_ESM.zip › Source Data/Figure 2/Figure 2b/Figure 2b.png]

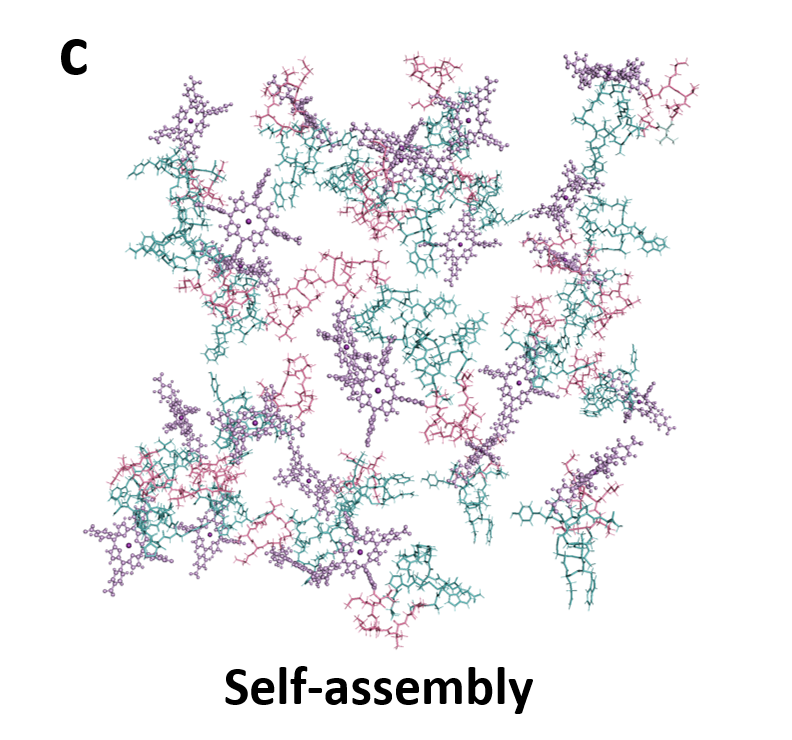

Supplement: Supplementary file 4 — Source Data [file 41467_2023_44665_MOESM4_ESM.zip › Source Data/Figure 2/Figure 2c/Figure 2c.png]

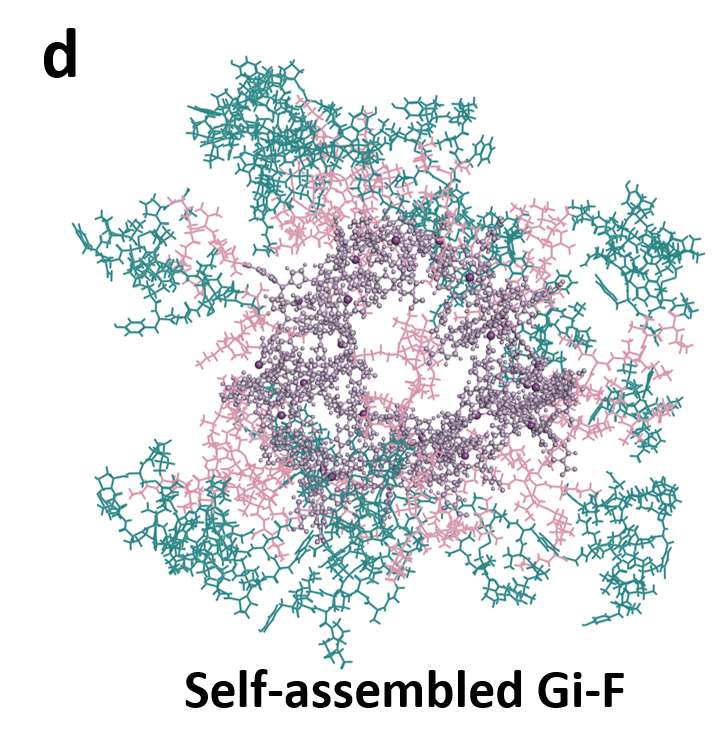

Supplement: Supplementary file 4 — Source Data [file 41467_2023_44665_MOESM4_ESM.zip › Source Data/Figure 2/Figure 2d/Figure 2d.png]

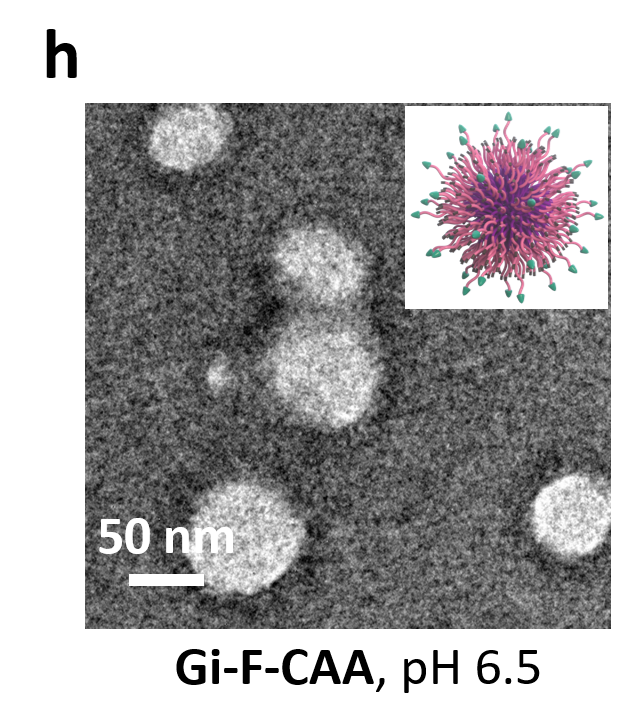

Supplement: Supplementary file 4 — Source Data [file 41467_2023_44665_MOESM4_ESM.zip › Source Data/Figure 2/Figure 2h/Figure 2h.png]

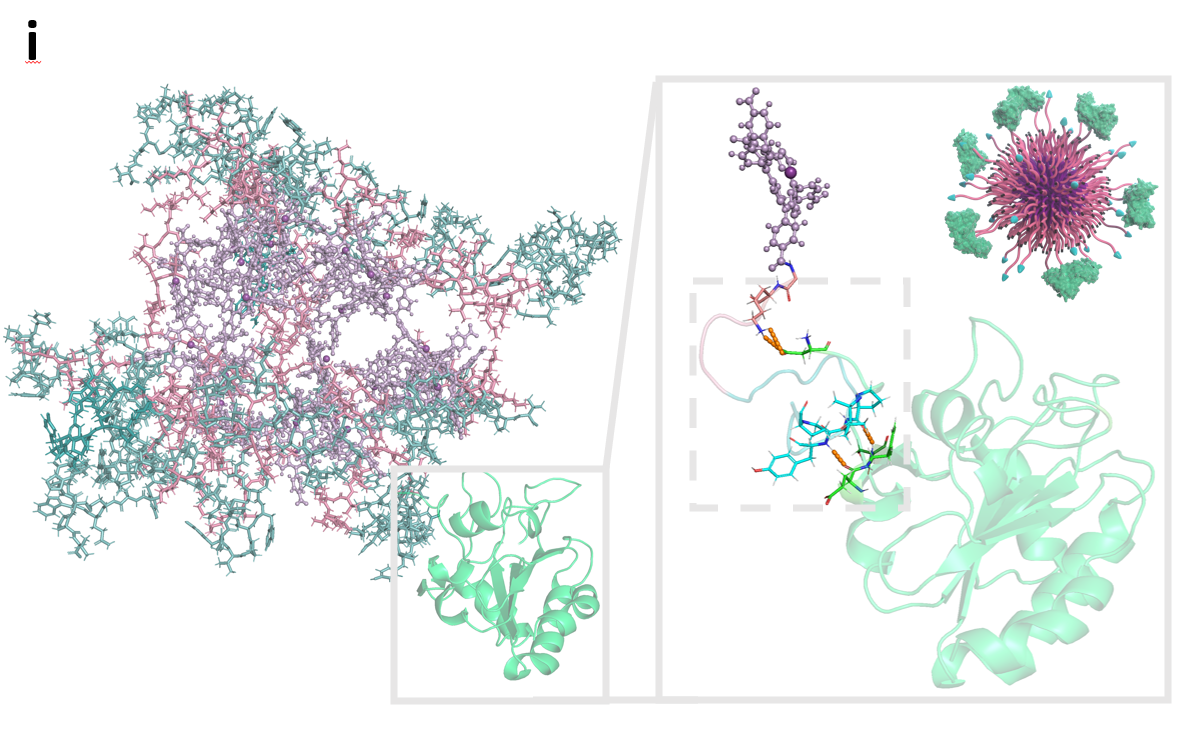

Supplement: Supplementary file 4 — Source Data [file 41467_2023_44665_MOESM4_ESM.zip › Source Data/Figure 2/Figure 2i/Figure 2i.png]

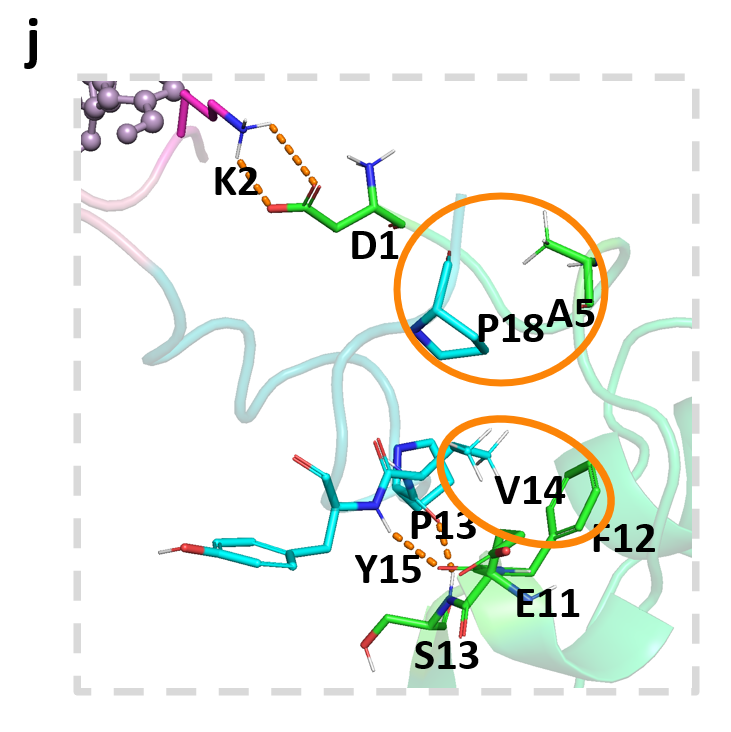

Supplement: Supplementary file 4 — Source Data [file 41467_2023_44665_MOESM4_ESM.zip › Source Data/Figure 2/Figure 2j/Figure 2j.png]

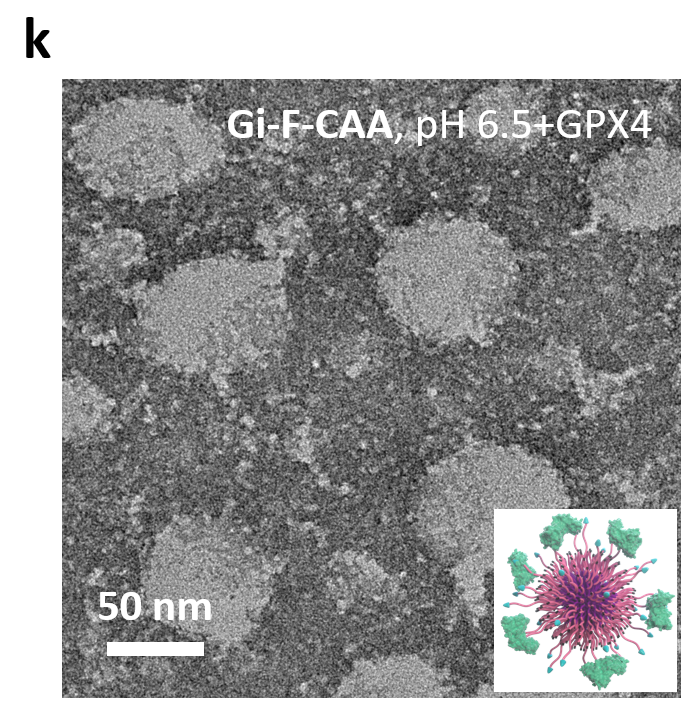

Supplement: Supplementary file 4 — Source Data [file 41467_2023_44665_MOESM4_ESM.zip › Source Data/Figure 2/Figure 2k/Figure 2k.png]

**a**

IHC of GPX4 in tumor

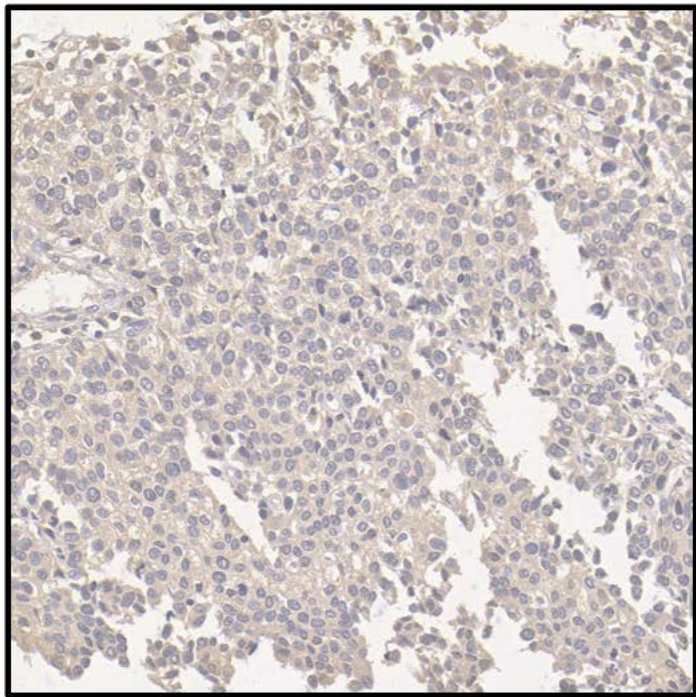

IHC of GPX4 in normal

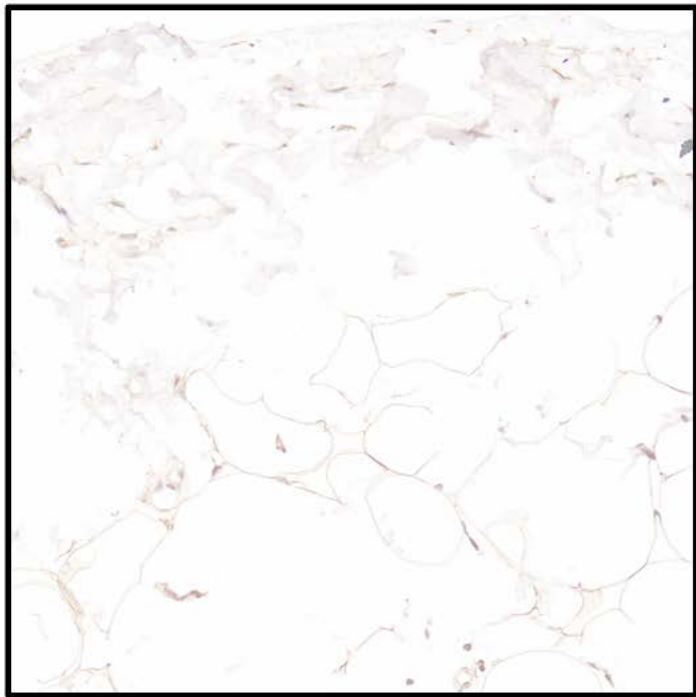

Supplement: Supplementary file 4 — Source Data [file 41467_2023_44665_MOESM4_ESM.zip › Source Data/Figure 3/Figure 3a/Figure 3a.pdf]

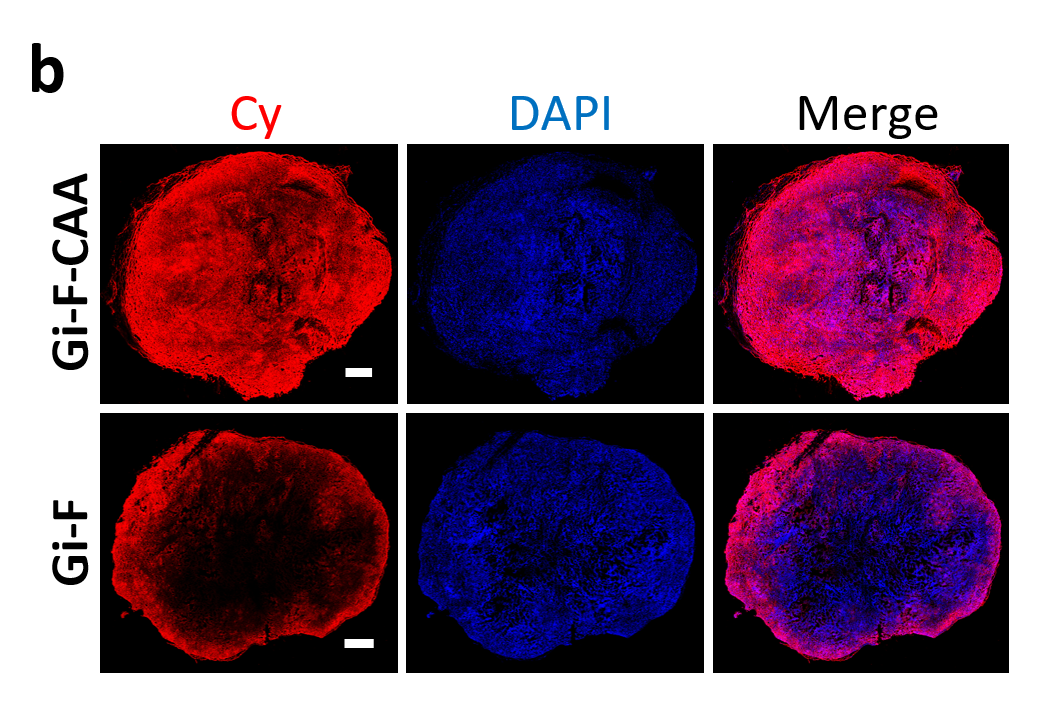

Supplement: Supplementary file 4 — Source Data [file 41467_2023_44665_MOESM4_ESM.zip › Source Data/Figure 3/Figure 3b/Figure 3b.png]

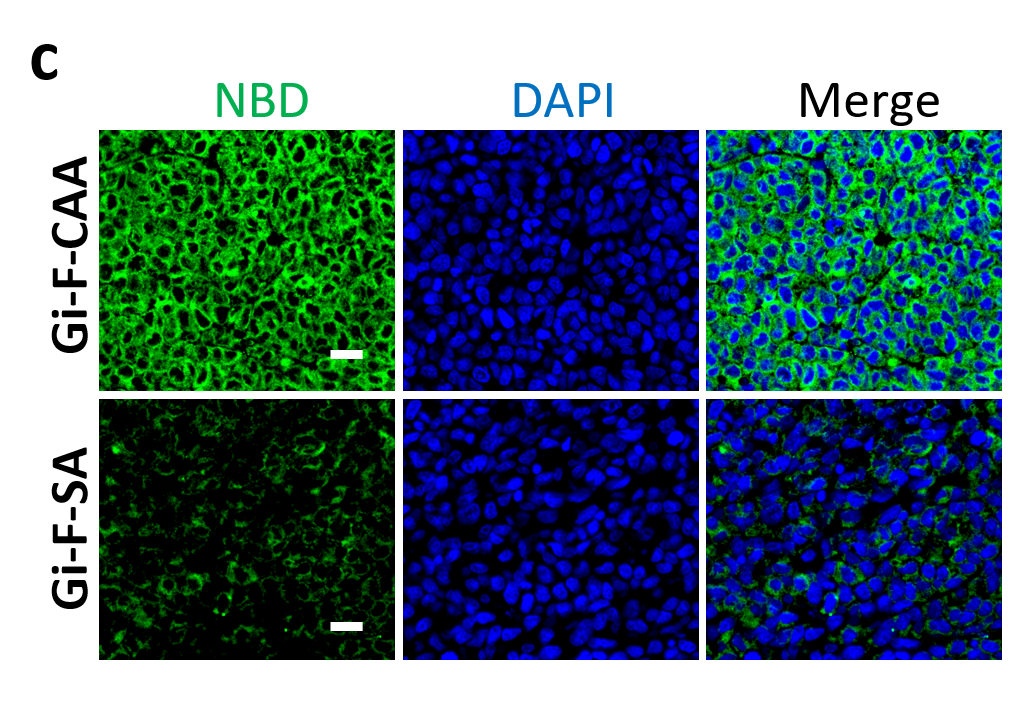

Supplement: Supplementary file 4 — Source Data [file 41467_2023_44665_MOESM4_ESM.zip › Source Data/Figure 3/Figure 3c/Figure 3c.png]

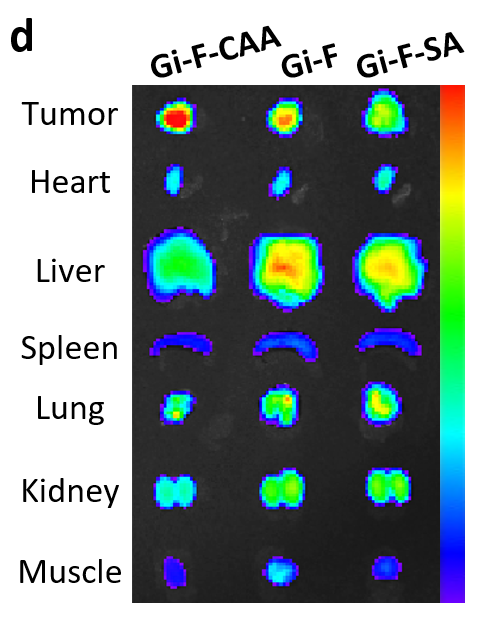

Supplement: Supplementary file 4 — Source Data [file 41467_2023_44665_MOESM4_ESM.zip › Source Data/Figure 3/Figure 3d/Figure 3d.png]

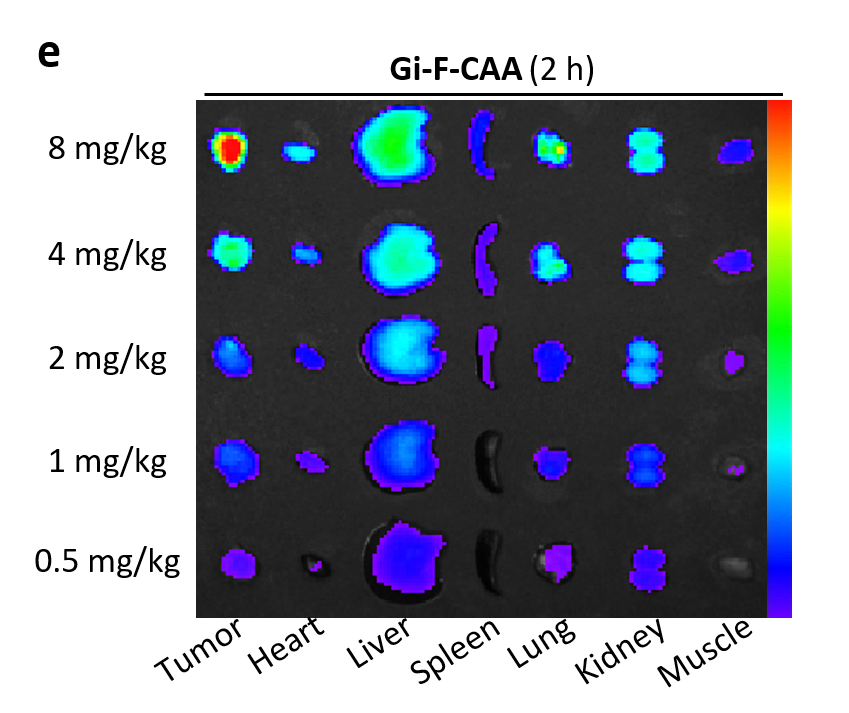

Supplement: Supplementary file 4 — Source Data [file 41467_2023_44665_MOESM4_ESM.zip › Source Data/Figure 3/Figure 3e/Figure 3e.png]

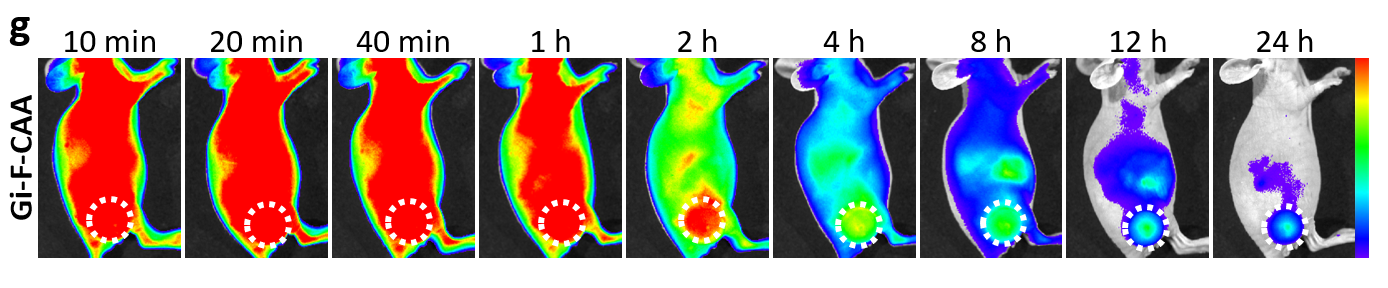

Supplement: Supplementary file 4 — Source Data [file 41467_2023_44665_MOESM4_ESM.zip › Source Data/Figure 3/Figure 3g/Figure 3g.png]

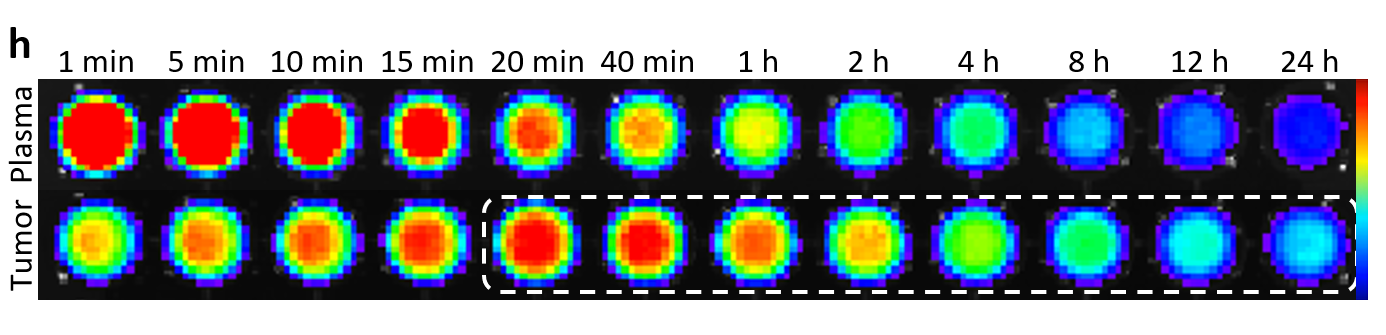

Supplement: Supplementary file 4 — Source Data [file 41467_2023_44665_MOESM4_ESM.zip › Source Data/Figure 3/Figure 3h/Figure 3h.png]

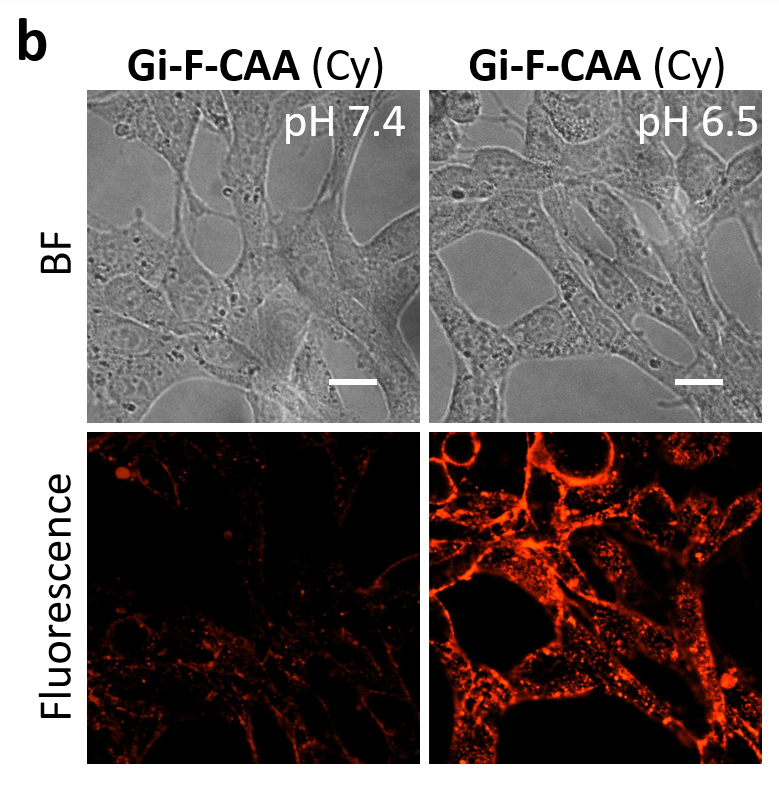

Supplement: Supplementary file 4 — Source Data [file 41467_2023_44665_MOESM4_ESM.zip › Source Data/Figure 4/Figure 4b/Figure 4b.png]

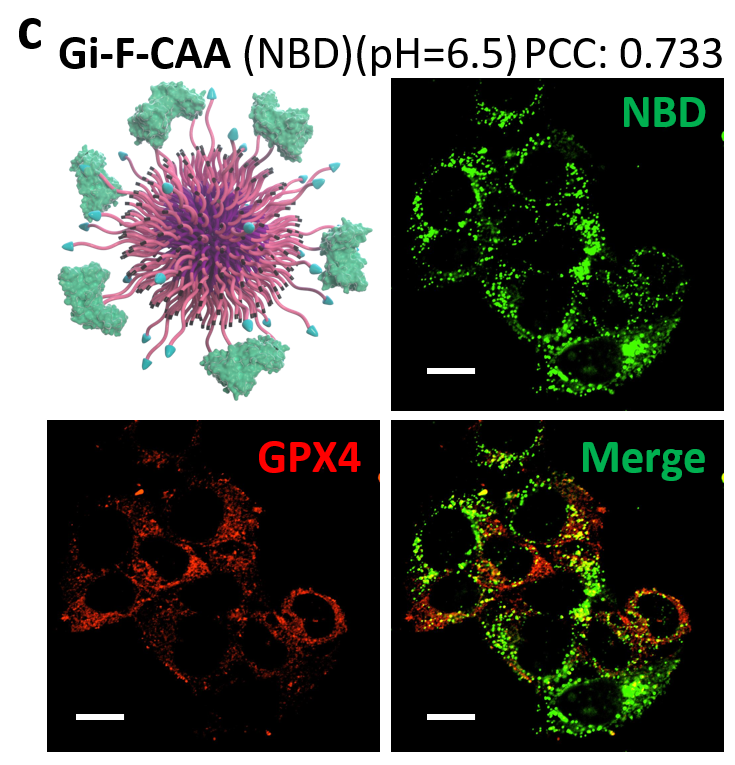

Supplement: Supplementary file 4 — Source Data [file 41467_2023_44665_MOESM4_ESM.zip › Source Data/Figure 4/Figure 4c/Figure 4c.png]

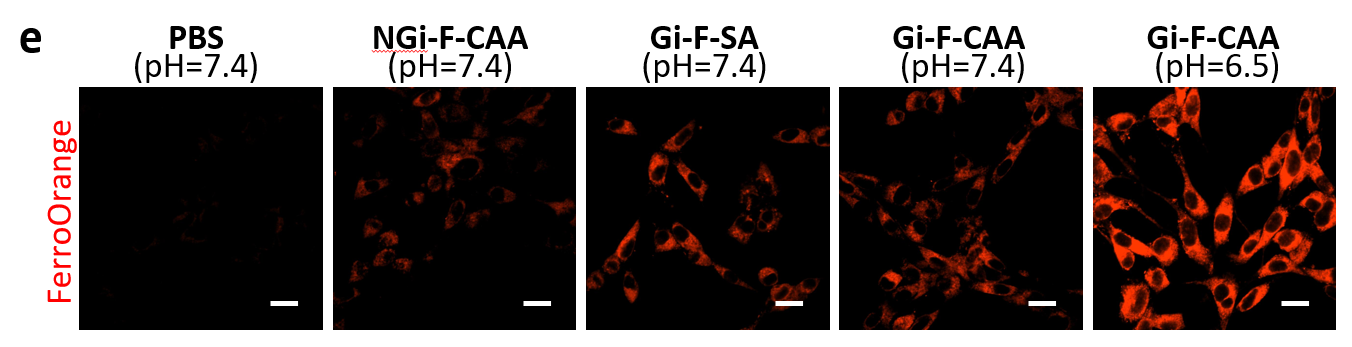

Supplement: Supplementary file 4 — Source Data [file 41467_2023_44665_MOESM4_ESM.zip › Source Data/Figure 4/Figure 4e/Figure 4e.png]

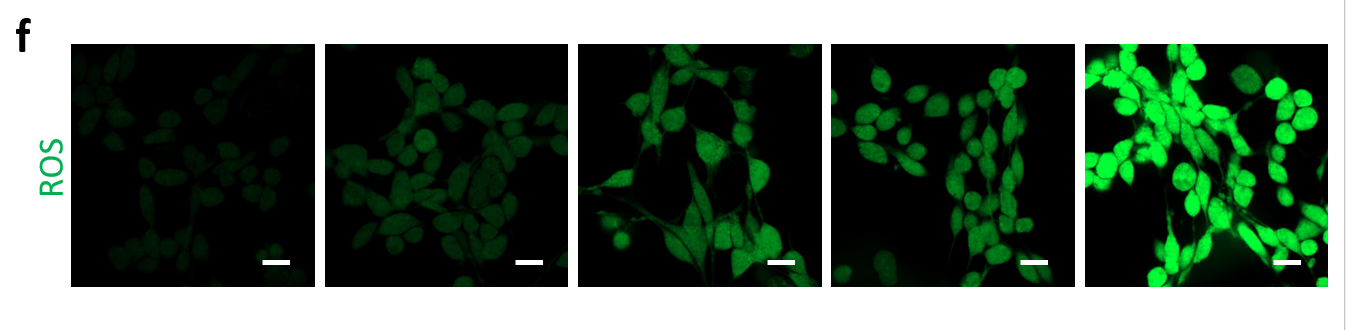

Supplement: Supplementary file 4 — Source Data [file 41467_2023_44665_MOESM4_ESM.zip › Source Data/Figure 4/Figure 4f/Figure 4f.png]

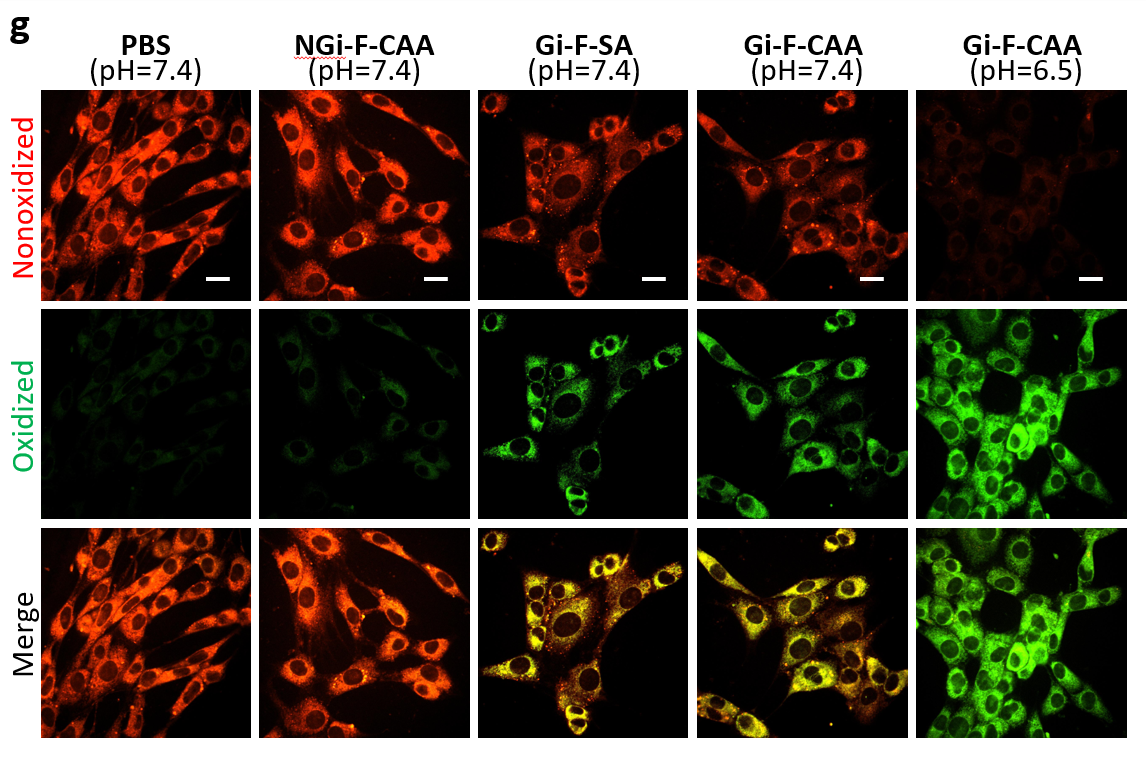

Supplement: Supplementary file 4 — Source Data [file 41467_2023_44665_MOESM4_ESM.zip › Source Data/Figure 4/Figure 4g/Figure 4g.png]

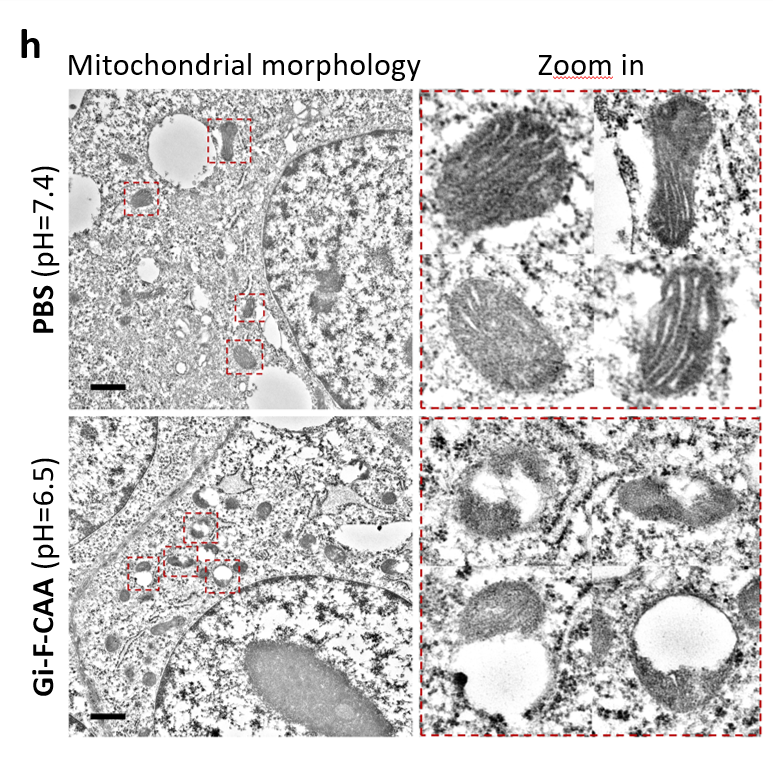

Supplement: Supplementary file 4 — Source Data [file 41467_2023_44665_MOESM4_ESM.zip › Source Data/Figure 4/Figure 4h/Figure 4h.png]

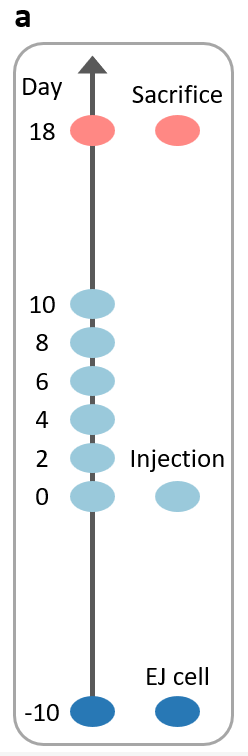

Supplement: Supplementary file 4 — Source Data [file 41467_2023_44665_MOESM4_ESM.zip › Source Data/Figure 5/Figure 5a/Figure 5a.png]

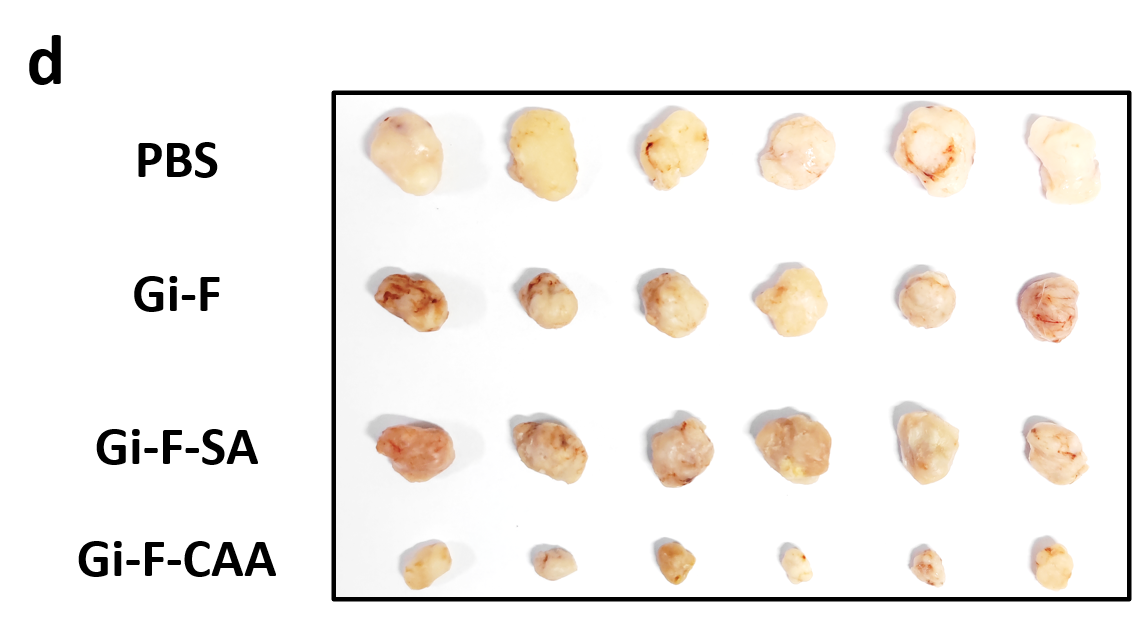

Supplement: Supplementary file 4 — Source Data [file 41467_2023_44665_MOESM4_ESM.zip › Source Data/Figure 5/Figure 5d/Figure 5d.png]

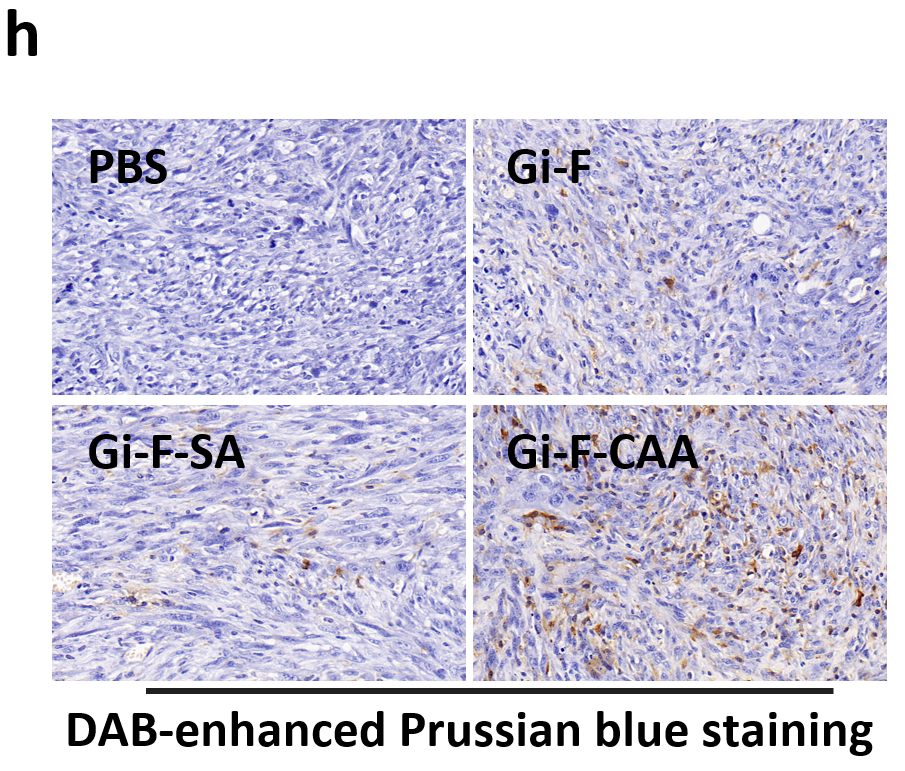

Supplement: Supplementary file 4 — Source Data [file 41467_2023_44665_MOESM4_ESM.zip › Source Data/Figure 5/Figure 5h/Figure 5h.png]

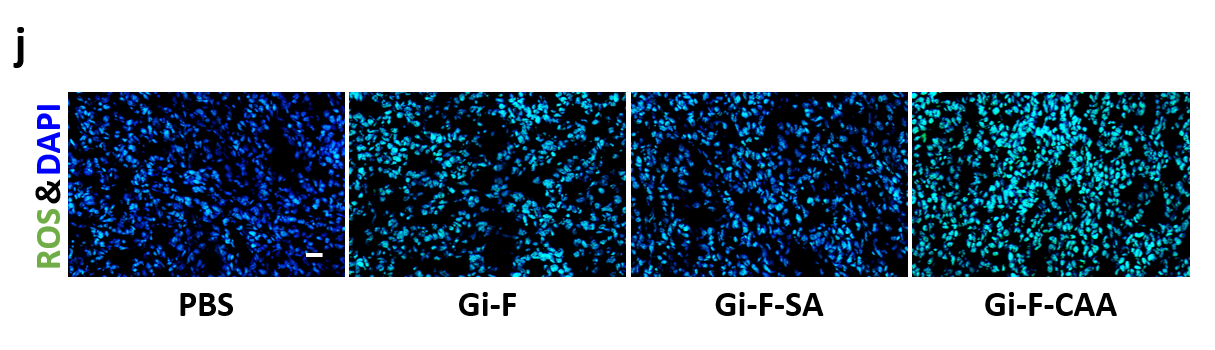

Supplement: Supplementary file 4 — Source Data [file 41467_2023_44665_MOESM4_ESM.zip › Source Data/Figure 5/Figure 5j/Figure 5j.png]

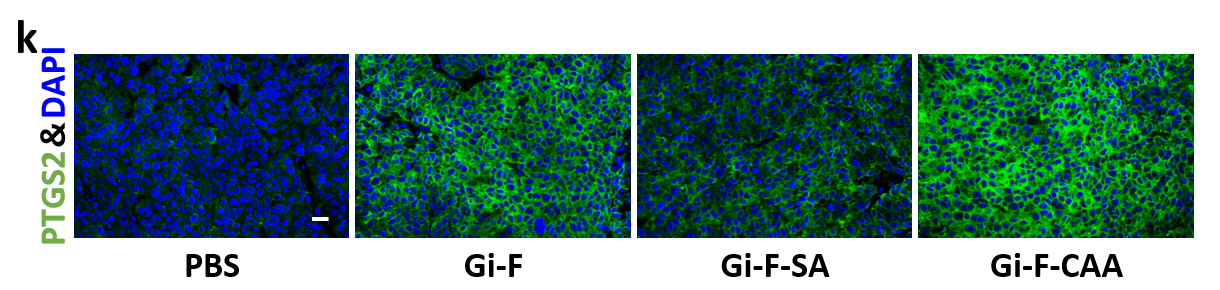

Supplement: Supplementary file 4 — Source Data [file 41467_2023_44665_MOESM4_ESM.zip › Source Data/Figure 5/Figure 5k/Figure 5k.png]

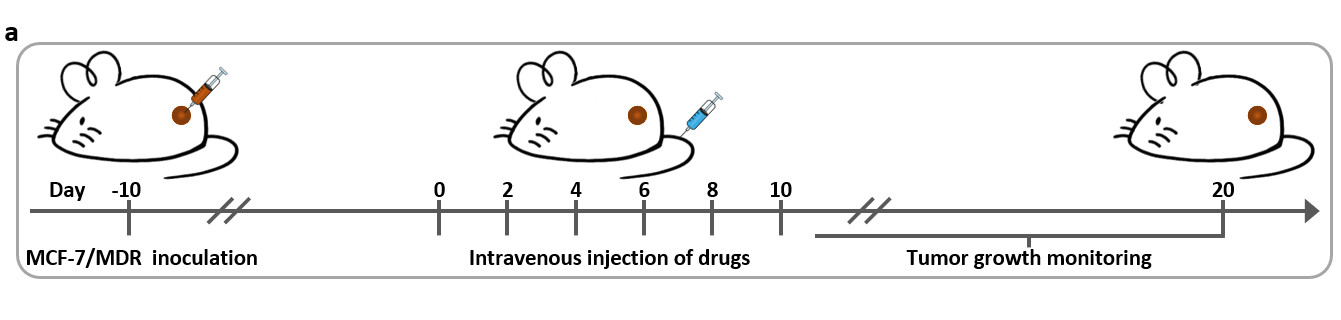

Supplement: Supplementary file 4 — Source Data [file 41467_2023_44665_MOESM4_ESM.zip › Source Data/Figure 6/Figure 6a/Figure 6a.png]

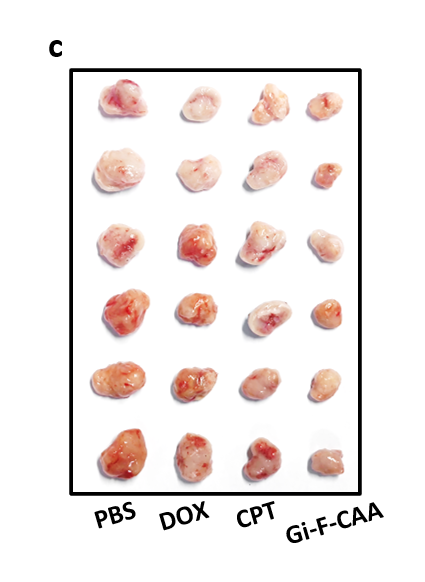

Supplement: Supplementary file 4 — Source Data [file 41467_2023_44665_MOESM4_ESM.zip › Source Data/Figure 6/Figure 6c/Figure 6c.png]

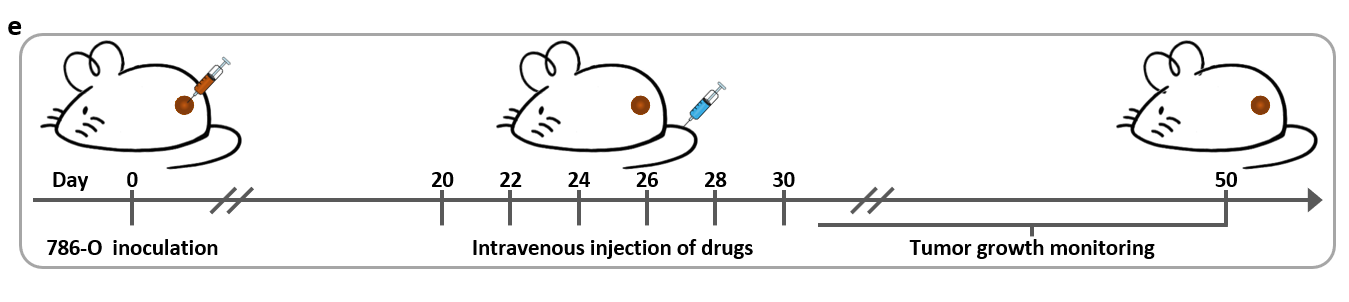

Supplement: Supplementary file 4 — Source Data [file 41467_2023_44665_MOESM4_ESM.zip › Source Data/Figure 6/Figure 6e/Figure 6e.png]

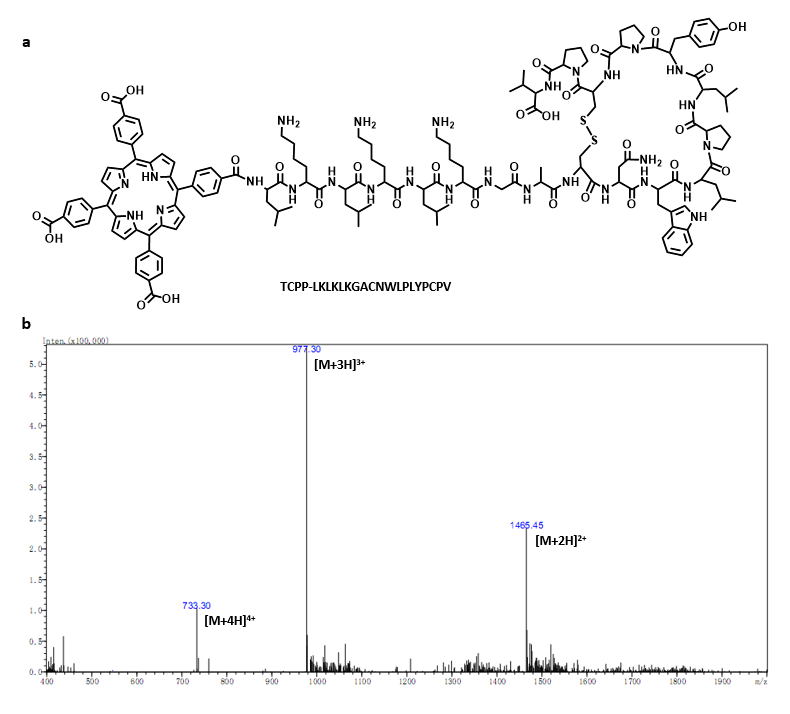

Supplement: Supplementary file 4 — Source Data [file 41467_2023_44665_MOESM4_ESM.zip › Source Data/Supplementary Figure 1/Supplementary Figure 1.png]

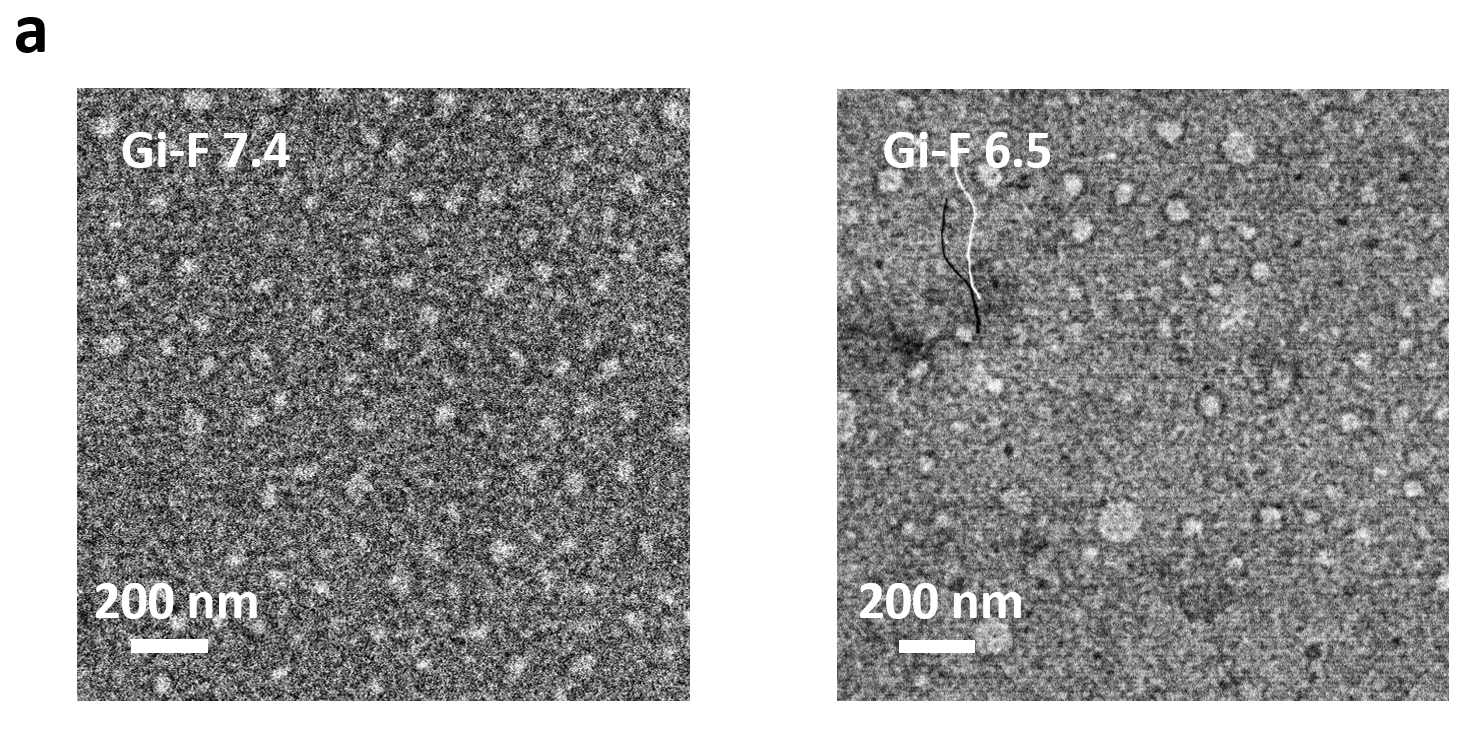

Supplement: Supplementary file 4 — Source Data [file 41467_2023_44665_MOESM4_ESM.zip › Source Data/Supplementary Figure 14/Supplementary Figure 14a/Supplementary Figure 14a.png]

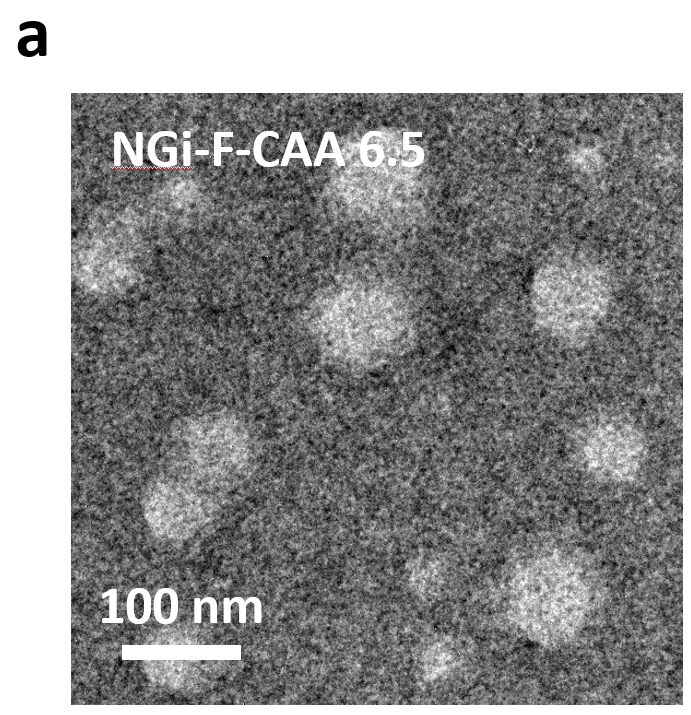

Supplement: Supplementary file 4 — Source Data [file 41467_2023_44665_MOESM4_ESM.zip › Source Data/Supplementary Figure 16/Supplementary Figure 16a/Supplementary Figure 16a.png]

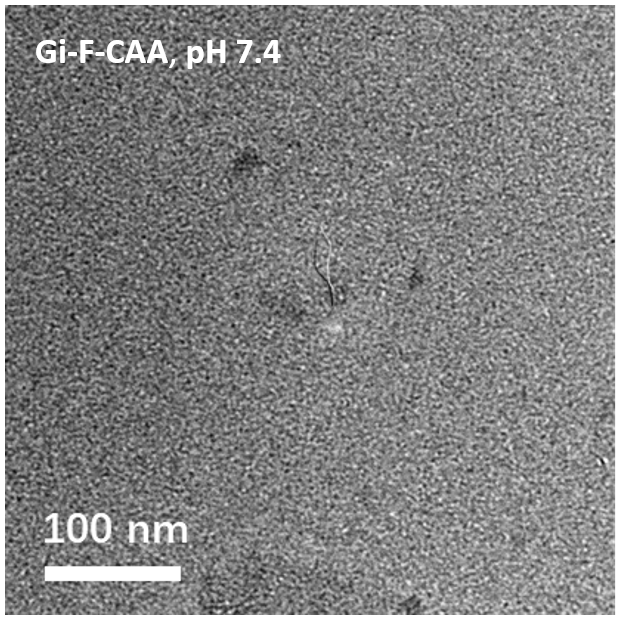

Supplement: Supplementary file 4 — Source Data [file 41467_2023_44665_MOESM4_ESM.zip › Source Data/Supplementary Figure 17/Supplementary Figure 17.png]

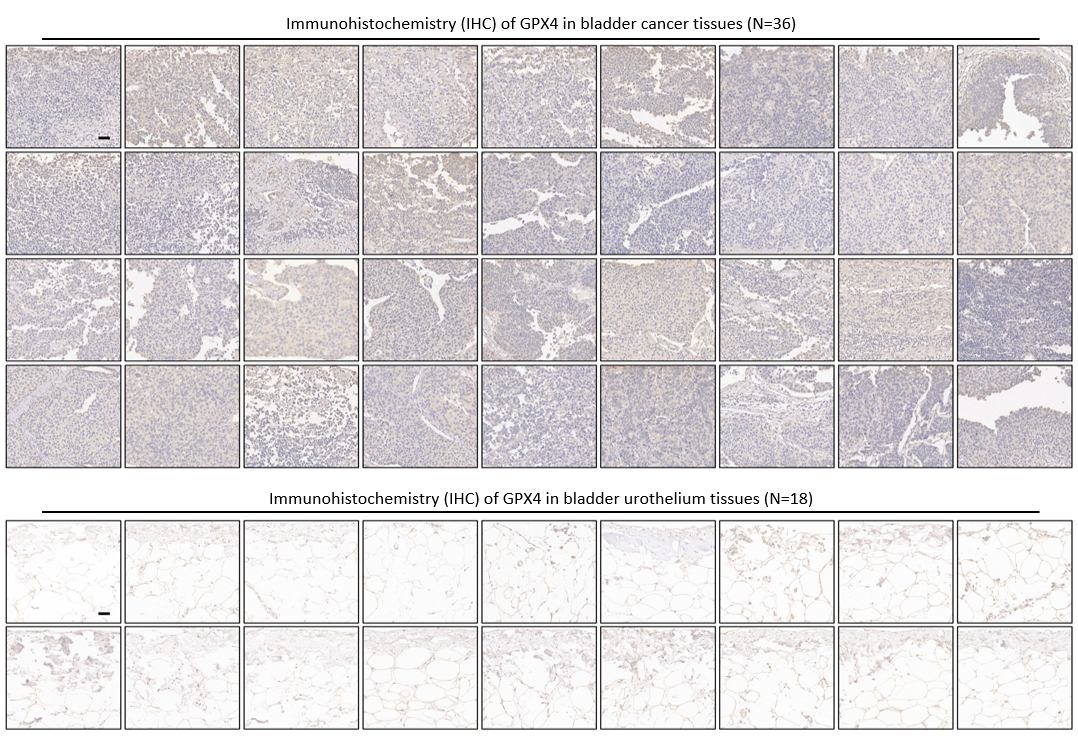

Supplement: Supplementary file 4 — Source Data [file 41467_2023_44665_MOESM4_ESM.zip › Source Data/Supplementary Figure 23/Supplementary Figure 23.png]

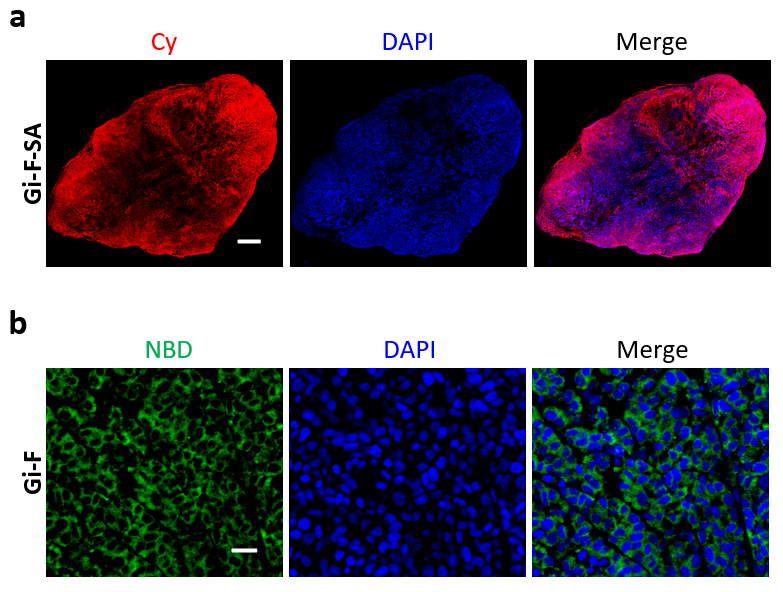

Supplement: Supplementary file 4 — Source Data [file 41467_2023_44665_MOESM4_ESM.zip › Source Data/Supplementary Figure 24/Supplementary Figure 24.png]

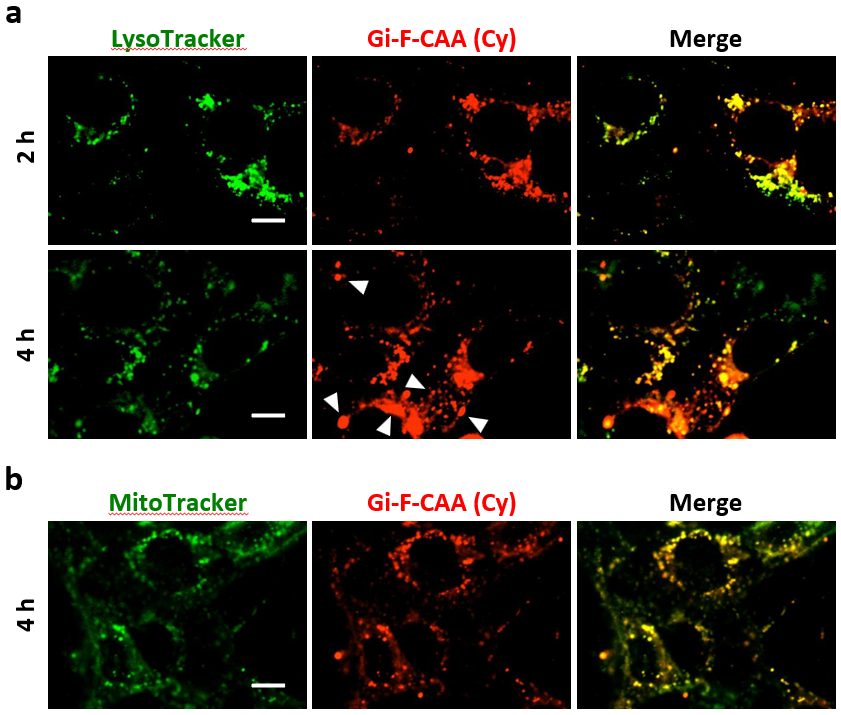

Supplement: Supplementary file 4 — Source Data [file 41467_2023_44665_MOESM4_ESM.zip › Source Data/Supplementary Figure 28/Supplementary Figure 28.png]

PBS

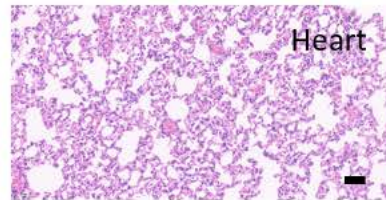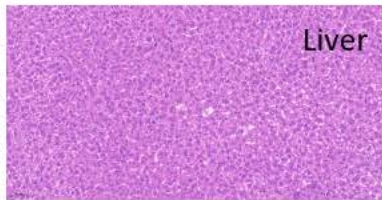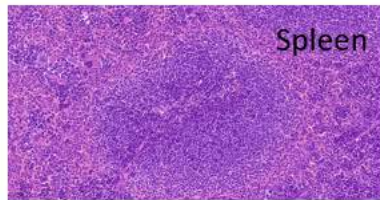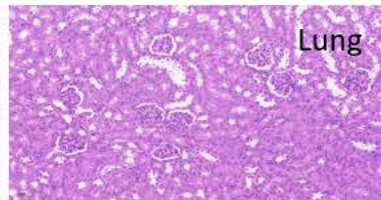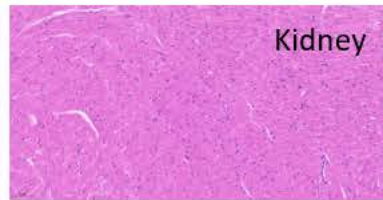

Gi-F-CAA  
(8 mg/kg)

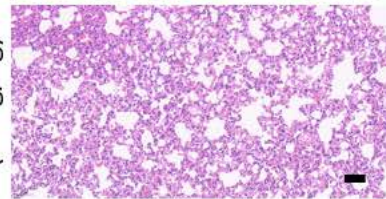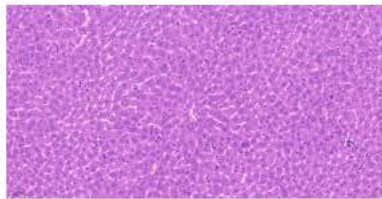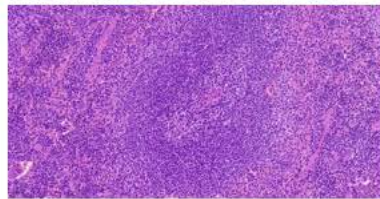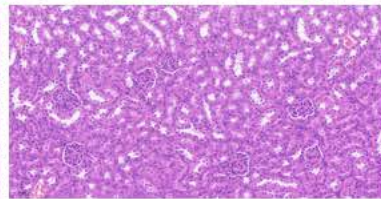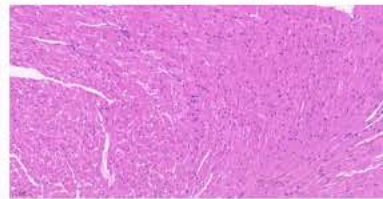

Gi-F-CAA  
(12 mg/kg)

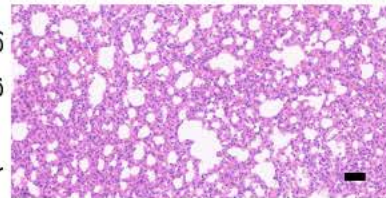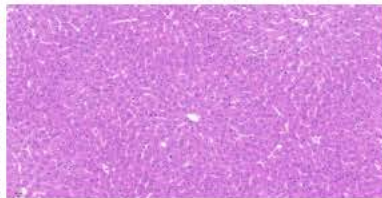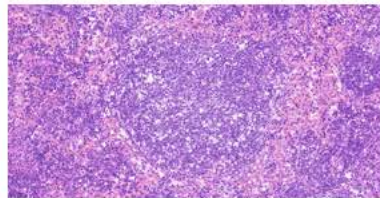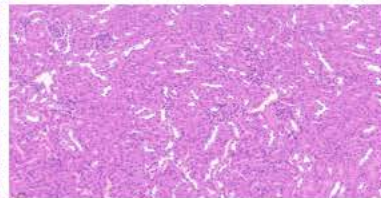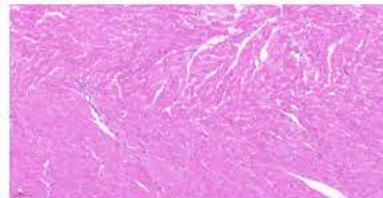

Gi-F-CAA  
(16 mg/kg)

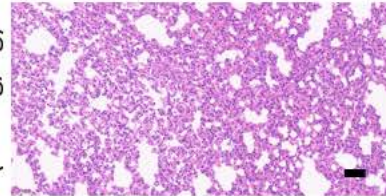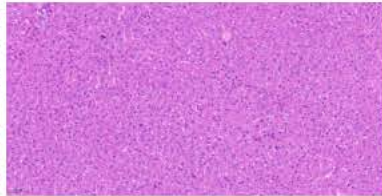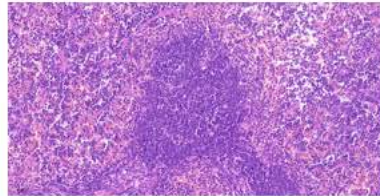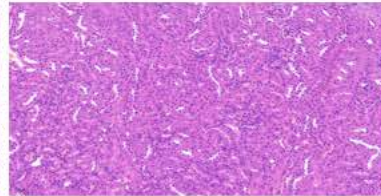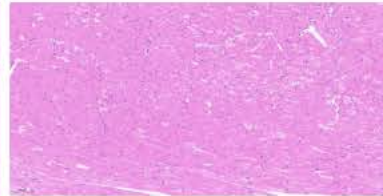

Supplement: Supplementary file 4 — Source Data [file 41467_2023_44665_MOESM4_ESM.zip › Source Data/Supplementary Figure 36/Supplementary Figure 36.pdf]

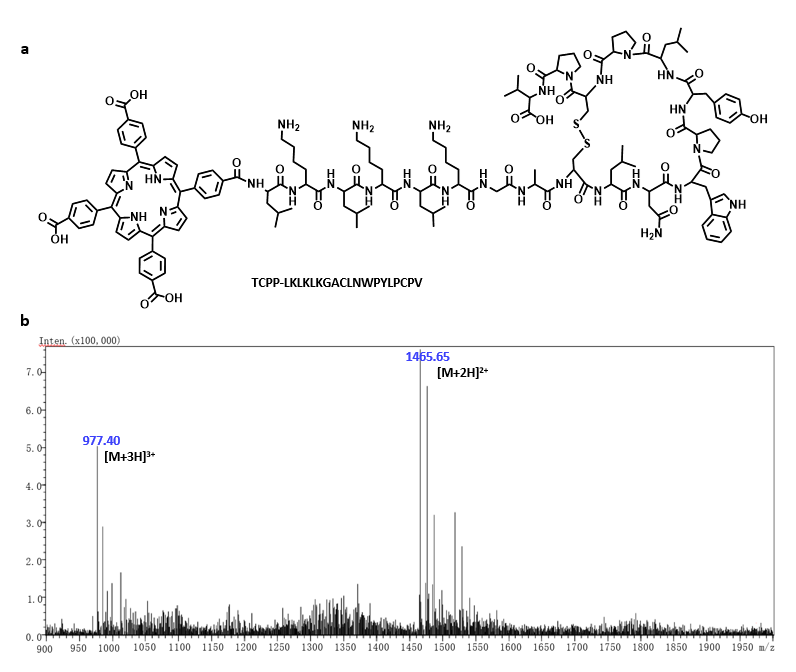

Supplement: Supplementary file 4 — Source Data [file 41467_2023_44665_MOESM4_ESM.zip › Source Data/Supplementary Figure 6/Supplementary Fig. 6.png]

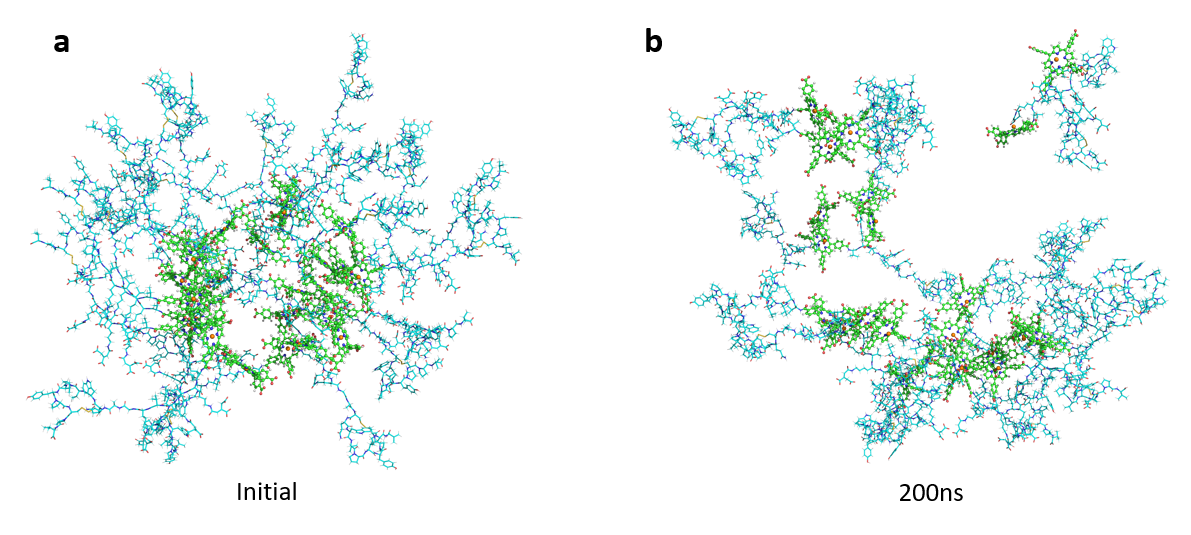

Supplement: Supplementary file 4 — Source Data [file 41467_2023_44665_MOESM4_ESM.zip › Source Data/Supplementary Figure 8/Supplementary Figure 8.png]
